# Role of phospholipase Cη1 in lateral habenula astrocytes in depressive-like behavior in mice

**DOI:** 10.1038/s12276-025-01432-1

**Published:** 2025-04-10

**Authors:** Sukwoon Song, Miseon Kang, Jiyoung Lee, Yong Ryoul Yang, Ho Lee, Jae-Ick Kim, Beomsue Kim, Hoon-seong Choi, Eun-bin Hong, Min-ho Nam, Pann-Ghill Suh, Jeongyeon Kim

**Affiliations:** 1 Emotion, Cognition and Behavior Research Group, Korea Brain Research Institute, Daegu, Republic of Korea; 2 Aging Convergence Research Center, Korea Research Institute of Bioscience and Biotechnology, Daejeon, Republic of Korea; 3 Cancer Experimental Resources Branch, National Cancer Center, Goyang, Republic of Korea; 4 Department of Biological Sciences, Ulsan National Institute of Science and Technology, Ulsan, Republic of Korea; 5 Neural Circuits Research Group, Korea Brain Research Institute, Daegu, Republic of Korea; 6 Research Animal Resource Center, Korea Institute of Science and Technology, Seoul, Republic of Korea; 7 Brain Science Institute, Korea Institute of Science and Technology, Seoul, Republic of Korea; 8 Department of Biotechnology, Yonsei University, Seoul, Republic of Korea; 9 Korea Brain Research Institute, Daegu, Republic of Korea; 10 Department of Brain Sciences, Daegu Gyeongbuk Institute of Science and Technology, Daegu, Republic of Korea

**Keywords:** Astrocyte, Long-term depression, Depression

## Abstract

Phospholipase C (PLC) enzymes play crucial roles in intracellular calcium-signaling transduction. Several brain PLC subtypes have been extensively studied, implicating them in psychiatric disorders such as depression, epilepsy and schizophrenia. However, the role of the recently identified PLCη remains largely unknown. We found that PLCη1 is prominently expressed in lateral habenula (LHb) astrocytes. Here, to investigate its physiological role, we generated astrocyte-specific PLCη1 conditional knockout (cKO) mice (*Plch1*^*f/f*^; *Aldh1l1-Cre*^*ERT2*^). In these cKO mice, we observed a reduction in cellular morphological complexity metrics, such as total process length, as well as a decrease in the passive membrane conductance of LHb astrocytes. Additionally, neuronal function was impacted by the cKO, as the synaptic efficacy and firing rates of LHb neurons increased, while extrasynaptic long-term depression was impaired. Both tonic α-amino-3-hydroxy-5-methyl-4-isoxazolepdlropionic acid receptor/*N*-methyl-d-aspartate receptor (AMPAR/NMDAR) currents and extracellular glutamate levels were reduced. Interestingly, chemogenetic activation of astrocytes restored the reduced tonic AMPAR/NMDAR currents in cKO mice. Furthermore, LHb astrocyte-specific deletion of PLCη1 via AAV-GFAP-Cre injection induced depressive-like behaviors in mice, which were reversed by chemogenetic activation of LHb astrocytes. Finally, we found that restraint stress exposure decreased *Plch1* mRNA expression in the LHb. These findings suggest that PLCη1 could be a potential therapeutic target for depression and highlight the critical role of astrocytes in the etiology of neuropsychiatric disorders.

## PLCη1 in astrocytes linked to depression and neuronal function

Phospholipase C (PLC) enzymes play a key role in brain cell communication. They help release calcium ions, which are important for brain functions such as memory and mood regulation. Problems with PLC signaling are linked to brain disorders such as depression and epilepsy. Here the authors explore the role of a specific PLC type, PLCη1, in astrocyte brain cells within a brain region known as the lateral habenula, using mice with a targeted deletion of PLCη1 in lateral habenula astrocytes to study its effects. Removing PLCη1 altered the shape and function of these astrocytes, leading to changes in neuron activity and behavior. The mice showed signs of depression, such as increased immobility in stress tests. The study also found that activating astrocytes could reverse these depressive behaviors.

This summary was initially drafted using artificial intelligence, then revised and fact-checked by the author.

## Introduction

Phospholipase C (PLC) enzymes are crucial for intracellular signaling molecules. They break down phosphatidylinositol 4,5-bisphosphate, leading to the release of second messengers such as inositol 1,4,5-trisphosphate (IP_3_) and diacylglycerol. IP_3_ triggers the release of Ca^2+^ from intracellular stores, increasing the cytosolic Ca^2+^ concentration^1–3^. This increase in intracellular Ca^2+^ can have profound implications for brain cells, influencing processes such as protein synthesis during long-term potentiation^4^, neurite outgrowth^5^, astrocytic neuromodulation and blood–vessel interactions^6^. Also, dysregulation of PLC signaling has been linked to several brain disorders^7^, including epilepsy^8^, schizophrenia^9,10^, bipolar disorder^11,12^, Huntington’s disease^13^, depression^14,15^ and Alzheimer’s disease^16^. The sixth class of PLC, PLC-eta (PLCη), was recently discovered in mammals^17,18^ and has two main subtypes, PLCη1 and PLCη2, both found in the brain^17,19^. It has been speculated that PLCη could be involved in brain functions including neural proliferation, synaptic plasticity and neuronal excitability^20–22^, and recent studies have shed light on the function of PLCη2 and its role in regulating neural outgrowth^23,24^. However, the specific biophysical roles of PLCη1 in the brain remain largely unexplored. Interestingly, a recent transcriptomic analysis in rat habenula found that *Plch1* expression is reduced after exposure to restraint stress^25^, providing a clue to the functional role of PLCη1 in the stress-related psychiatric model.

The lateral habenula (LHb) is a small brain structure located in the epithalamus and activated by the limbic forebrain in response to aversive stimuli or the absence of expected reward stimuli^26^. Such activation has been linked to the regulation of depressive states by recent studies^27–29^. Hyperactivity in the LHb has been demonstrated in several animal models of depression and a growing body of evidence further supports an association between LHb activities and depression^29,30^. Notably, previous studies have indicated that habenular express PLCη1 (refs. ^17,31^); however, the specific role of PLCη in the LHb regarding depression remains unexplored.

Astrocytes are the most common type of glial cells in the brain. They have a star-shaped morphology and intricate processes extending outward, forming extensive networks that facilitate the interaction between neurons or epithelial cells of blood vessels. They support the blood–brain barrier^32^, provide nutrients to nervous tissues^33^ and repair the brain and spinal cord following infection and traumatic injuries^34^. Another essential function of astrocytes is spatial buffering, a process that restricts extracellular K^+^ levels by absorbing K^+^ through the inwardly rectifying potassium channel 4.1 (Kir4.1)^35^. When Kir4.1 activity is compromised, accumulated extracellular K^+^ can increase neuronal excitability and induce epilepsy^35,36^. In addition, recent evidence suggests that astrocytes mediate synaptic plasticity through gliotransmission, often in an astrocyte Ca^2+^ activity-dependent manner^37–40^, and that their dysfunction may contribute to the pathophysiology of mood disorders, including depression^41–44^. Upregulation of the Kir4.1 has been associated with LHb neuronal firing modes in rat models of depression^45^, implying that glial control of LHb neurons is critical for mediating mood in mammals. PLCη1 may be primarily expressed in astrocytes among various cell types^46^, which makes the molecule a candidate for targeted medical intervention.

In this study, we aimed to investigate the role of PLCη1 in astrocytes within the LHb and its implications in depression. We used mouse models with the astrocyte-specific deletion of PLCη1 in the LHb to evaluate its physiological impacts on astrocytes, neuronal functions and behavioral changes. It is noteworthy that this study explores the role of PLCη1 concerning brain function, and our findings will provide valuable insights into the broader molecular mechanisms underlying depression and other mood disorders.

## Materials and methods

### Animals

To generate *Plch1*^*f/f*^ mice, we used the KOMP ES cell line *Plch1*^*tm1a(KOMP)Wtsi*^ (RRID: MMRRC_060360-UCD) from the Mutant Mouse Resource and Research Center at the University of California at Davis, an National Institutes of Health (NIH)-funded strain repository donated to the Mutant Mouse Resource and Research Center by the KOMP Repository, University of California, originating from Pieter de Jong, Kent Lloyd, William Skarnes, Allan Bradley, Wellcome Sanger Institute. F1 mice were crossed with flipperase transgenic mice to eliminate the FRT-Neo cassette. The *Plch1*^*f/f*^ mice, originally on a mixed 129 × C57BL/6 background, were backcrossed with C57BL/6 mice for at least eight generations before the experiments. *Plch1*^*f/f*^ mice were genotyped using a forward primer sequence (5′ to 3′) of GCA TTA AGA CTA GGT TGC TCC TG and a backward primer sequence (5′ to 3′) of AAC CAT CAG TGT TTC ACC AGC TC. *Aldh1l1-Cre*^*ERT2*^ mice^47^ were purchased from the Jackson Laboratory (RRID: IMSR_JAX:029655). To investigate the effects of astrocyte-specific PLCη1 deletion, *Plch1*^*f/f*^ and *Aldh1l1-Cre*^*ERT2*^ mice were intercrossed to produce *Plch1*^*f/f*^; *Aldh1l1-Cre*^*ERT2*^ mice. At age 6–8 weeks, mice were given intraperitoneal (i.p.) injections of tamoxifen (10 mg/kg body weight, 0.01 g/ml in sunflower seed oil) for five consecutive days and were then used for experiments. For LHb astrocyte-specific gene deletion in the LHb, an AAV-GFAP-Cre-mCherry viral vector was microinjected into the LHb of 3-month-old *Plch1*^*f/f*^ mice. The procedure for microinjection is described in detail in the subsequent surgery section. For experiments using *Plch1*^*f/f*^; *Aldh1l1-Cre*^*ERT2*^ mice, *Plch1*^*f/f*^ mice that received the same tamoxifen treatment were used as controls (CTLs). The mice were maintained on a 12 h light–dark cycle in a specific pathogen-free facility with a controlled temperature (21 ± 1 °C) and humidity (60%). The mice were housed in standard cages with unrestricted access to water and food. Only male mice were used in all experiments, except for western blot experiments, in which both sexes were used. All animal experiments were conducted under the guidelines of the Institutional Animal Care and Use Committee of the Korea Brain Research Institute.

### Surgery

To suppress PLCη1 expression in LHb astrocytes, 0.5 µl adeno-associated viral (AAV) vectors (AAV-GFAP-Cre-mcherry; for the CTL, AAV-GFAP-mCherry) were bilaterally injected into the LHb of *Plch1*^*f/f*^ mice. For chemogenetic activation of LHb astrocytes in *Plch1*^*f/f*^; *Aldh1l1-Cre*^*ERT2*^ mice (for the patch-clamp experiment, 0.5 µl AAV-DIO-hM3Gq-mCherry; for the CTL, AAV-DIO-mCherry) were injected bilaterally. For suppressing PLCη1 expression and chemogenetic activation of LHb astrocytes, 0.6 µl of 1:1 cocktail (AAV-GFAP-Cre and AAV-DIO-hM3Gq-mCherry/AAV-DIO-mCherry) of viral vector was delivered bilaterally. Mice were anesthetized with Avertin (250 mg/kg) and positioned in a stereotactic frame. Blunt needles (33 gauge, Nanofil, 1209K, WPI) were placed at the coordinates of medial-lateral (ML) ±0.5 mm, antero-posterior (AP) ±1.4 mm and dorso-ventral (DV) −2.9 mm from bregma. Titers of all virus used were ~5 × 10^12^ units/µl and injected at a rate of 0.2 µl/min. Blunt needles remained for 10 min after microinjection before retraction. Care was taken to maintain the temperature of the mice with a heating mat during the surgery. After surgery, the mice recovered for 2–3 weeks.

### Astrocyte culture

Primary cortical astrocytes were cultured from P0 to P3 *Plch1*^*f/f*^ mouse pups. The cerebral cortex was dissected without the meninges and gently triturated into single-cell units. The culture media were prepared as follows: Dulbecco’s modified Eagle’s medium (Invitrogen) was supplemented with 25 mM glucose, 2 mM glutamine, 10% heat-inactivated horse serum, 10% heat-inactivated fetal bovine serum and 1,000 units/ml penicillin–streptomycin. On the third day of culture, the cells were rinsed repeatedly with gentle pipetting, and the medium was replaced to remove debris. Cultured cells were maintained at 37 °C in a humidified 5% CO_2_ incubator. At 7 days in vitro, a subculture was carried out, seeded on three 60 Pi dishes, and kept in a cell culture incubator at 37 °C. Seven days after subculturing, the cells were infected with AAV. Cultured cells were maintained at 37 °C in a humidified incubator in a 5% CO_2_ atmosphere until they were used for RNA extraction experiments at 14 days in vitro.

### Western blot

The LHb of *Plch1*^*f/f*^; *Aldh1l1-Cre*^*ERT2*^ mice were dissected and lysed with lysis buffer (N-PER (Thermo Fisher, 87792) and Pierce Protease Inhibitor Mini Tablets, EDTA free (Thermo Fisher, a32955)). Lysates with loading buffer (4× Laemmli Sample Buffer (Bio-Rad, 1610747) and β-mercaptoethanol (Sigma, M3148)) were boiled for 5 min and then centrifuged at 4 °C. This loading sample was subjected to sodium dodecyl-sulfate–polyacrylamide gel electrophoresis (SDS–PAGE) and transferred to a polyvinylidene difluoride (PVDF; Bio-Rad, 1620175) membrane. Membranes were blocked for 40 min with 5% skim milk in PBS containing 0.1% Triton X-100. For the detection of PLCη1, PLCη1 (Abcam, ab97751 or Proteintech, #9143-1-AP) and GAPDH (Abcam, ab181602) protein-specific antibodies were incubated with the membranes overnight at 4 °C. Membranes were then incubated with secondary antibodies (goat anti-Rabbit IgG CiteAb, A120-101P) for 1.5 h. Specific signals linked to horseradish peroxidase (HRP) at a dilution of 1:10,000 were visualized by enhanced chemiluminescence.

### RNA extraction and qRT–PCR

*Plch1* expression levels were analyzed using qRT–PCR. Cultured astrocytes were collected to extract RNA. According to the manufacturer’s protocol, total RNA was extracted from the pooled samples using carrier RNA from a few samples (Rneasy Micro Kit, Qiagen, 74004). The cDNA was synthesized using the Reverse Transcription 5× Premix (random hexamer, ELPIS-Biotech, EBT-1514) for 60 min at 42 °C. qRT–PCR was performed using an RT-PCR 7500 Fast system (Applied Biosystems) and RT–qPCR 2× Master Mix with SYBR Green and ROX dye (ELPIS-Biotech, EBT-1802). The PCR cycling parameters were 3 min at 94 °C, followed by 40 cycles of 10 s at 94 °C, 10 s at 55 °C and 30 s at 72 °C. Beta-actin was used for normalization, and the relative expression was calculated. The following oligonucleotide pairs were used: PLCη1 forward primer sequence (5′ to 3′), GTC TAC CTG GAA GGA CTC ACA G; PLCη1 reverse primer sequence (3′ to 5′), CTG AAG AAG CGT GCC TGG GAT T; Kcnj10 (Kir4.1) forward primer sequence (5' to 3'), GTC GGT CGC TAA GGT CTA TTA CA; Kcnj10 (Kir4.1) reverse primer sequence (3' to 5'), GGC CGT CTT TCG TGA GGA C; Eaat1 (GLAST) forward primer sequence (5' to 3'), GGG TTT TCA TTG GAG GGT TGC; Eaat1 (GLAST) reverse primer sequence (3' to 5'), CCA CGG GTT TCT CTG GTT CAT; Eaat2 (GLT-1) forward primer sequence (5' to 3'), GGG TCA TCC TGG ATG GAG GT; Eaat2 (GLT-1) reverse primer sequence (3' to 5'), CGT GTC GTC ATA AAC GGA CTG; β-actin forward primer sequence (5′ to 3′), GAG CTA TGA GCT GCC TGA CGG; and β-actin reverse primer sequence (3′ to 5′) CAG CTC AGT AAC AGT CCG CCT. All oligonucleotides were synthesized using BIONICS.

### FISH analysis

#### Sample pretreatment

For fluorescent in situ hybridization (FISH), mixed frozen brain slices (coronal section, 15 μm) were prepared with super adhesion glass slides (SuperFrost Plus slides, Epredia, J1800AMNZ). All slides were removed from the 100% ethanol and baked at 60 °C in a dry oven for 30 min. This step improves the sample attachment to the slides and avoids tissue removal from the slides during subsequent washing steps. After baking the slides to create a hydrophobic barrier around the sample, an Immedge hydrophobic barrier pen (Vector Laboratories) was used to avoid touching or disrupting the tissue section. Utilizing the multiplex fluorescence V2 RNAscope kit (RNAscope Intro Pack for Multiplex Fluorescent Reagent Kit v2, ACD_323280), hydrogen peroxide and Protease III were applied according to the manufacturer’s instructions. As it was observed that a 30 min Protease III treatment caused overdigestion of the leaf sections, the treatment was reduced to 15 min.

#### Probing FISH

The RNAscope assay was conducted by multiple hybridization steps (probe and AMP binding) and signal development (HRP channel binding), as described in the manufacturer’s protocol (RNAscope Intro Pack for Multiplex Fluorescent Reagent Kit v2 with TSA Vivid Dyes, ACD_323280, ACDBio). All probes were customized by ACDBio Corporation. The PLCη1 probe was customized in the C3 channel (Mm-Plcη1-C3, 556241-C3), and the S100β (Mm-S100β-C2, 431731-C2) and NeuN (Mm-RBfox3-C2, 313311-C2) probes were customized in the C2 channel. Single probes and multiplexed assays were used to validate the efficiency of the assays. The hybridizations were performed and analyzed using slides containing multiple independent tissue sections. For probe hybridization, the samples were incubated in the HybEz II humidifying system (HybEz Oven, 321721) for 3 h at 40 °C. In our experience, extended probe hybridization times of up to 4 h with the fixed-frozen samples improve the probe hybridization and subsequent visualization. The following day, slides were washed in 1× wash buffer, and AMP signal amplification and HRP-labeled probe hybridization steps were carried out according to the manufacturer’s protocol (Multiplex fluorescence V2 kit, ACDBio). We used the TSA Vivid fluorophore 520 (green) and fluorophore 570 (orange), which were diluted at 1:1,500 to visualize the Mm-Plcη1-C3 signal in green, Gfap-C2 signal and Mm-RBfox3(NeuN)-C2 signal in orange. Then the TSA vivid fluorophore 650 was diluted at 1:3,000 to visualize the Mm-S100β-C2 signal in magenta and developed sample slides after AMP staining were treated with 4,6-diamidino-2-phenylindole (DAPI) nuclear stain. DAPI was removed by flicking the slides within 30 s, and a cover slip was placed using Everbright hard-set mounting medium. The slides were stored overnight in the dark before visualization for complete drying of hard-set mounting media (ProLong Gold Antifade Mount, Invitrogen, P36930).

### IHC

#### Sample preparation for IHC

The mice were anesthetized with Avertin (2,2,2-tribromoethanol, Sigma-Aldrich, T48402-100G) (250 mg/kg, i.p. injection). The brain was fixed with ice-cold 4% paraformaldehyde and saline through cardiac perfusion and removed from the skull. The whole brain was then consecutively incubated in 4% paraformaldehyde solution (overnight in a 4 °C cold room) and 30% sucrose (Sigma-Aldrich, 84097-500G) solution (dissolved in PBS for 3 days in a 4 °C cold room). Individual tissue blocks were embedded in OCT cryoprotectants (Sakura, 4583). For immunolabeling staining, cryoprotected coronal sections were serially obtained at 35 µm using a cryotome (Leica, CM1860).

#### Free-floating IHC

Immunohistochemistry (IHC) was performed using the standard free-floating method. The brain slices were washed twice for 10 min with 1× PBS solution (10× PBS; Biosesang, PR4007-100-00). Brain slices were permeabilized by a detergent-containing blocking solution for 1.5 h at room temperature (22–24 °C). The blocking solution contained 4% normal goat serum (Jackson ImmunoResearch Laboratories, 005-000-121), 0.5% bovine serum albumin (Bovine Fraction V; Thermo-Scientific, J10857-22) and 0.2% Triton X-100 (Sigma-Aldrich, T8787-250ML) in PBS. Primary antibodies were incubated overnight in a 4 °C cold room for immunolabeling staining. The primary antibody used was an anti-GFAP antibody (Millipore, Ab5541, 1:500). The secondary antibody used was goat anti-chicken IgG Alexa Fluor 488 (Invitrogen, A-11039, 1:500) for 1.5 h at room temperature. After three washes with PBS (15 min), the brain slices were mounted with a DAPI-containing mounting medium (VECTASHIELD HardSet Antifade Mounting Medium with DAPI, Vector Laboratories). Images were acquired using a slide scanner (3DHISTECH, Panoramic Scan) and a confocal microscope (Nikon Eclipse Ti-RCP).

### Image analysis

We utilized IMARIS software version 9.0 (Oxford Instruments) for three-dimensional (3D) reconstruction and quantitative analysis of IHC and in situ hybridization (ISH) images. Analytical parameters were defined based on established literature^48^, specifically tailored for astrocyte analysis. The Surface module was employed to detect astrocyte surfaces in the IHC study and cellular nuclei in the ISH study. Astrocyte surfaces were delineated using markers specific to astrocytic proteins, while cellular nuclei were reconstructed based on DAPI signals. Each reconstructed surface object corresponding to DAPI staining was presumed to represent a single nucleus. Astrocyte morphological features were quantified using the Filament module, which traced and measured the 3D structure of astrocytic processes. Parameters such as process length, branching points and total filament volume were extracted to assess astrocyte morphology comprehensively. The Spot module was used for the precise detection of mRNA probe puncta in ISH studies. A cell was classified as *Plch1*- or *S100b*-positive if mRNA probe puncta were located within a 1.5 µm radius of the reconstructed nuclear surface. This threshold distance was selected to minimize false-positive or false-negative associations.

### Behavior tests

#### TST

The tail suspension test (TST) was performed using the method described previously^49,50^. The mouse undergoing testing was secured on the acrylic cylindrical stick using tape in a visually isolated area (63 × 58 × 13 cm, Plexiglas box). Video tracking was employed to monitor the movement over 6 min, with data from the final 5 min utilized for immobile behavior analysis. Immobility was defined as the absence of any bodily movements, except struggling and swing behavior.

#### FST

For the forced swimming test (FST), the apparatus was a glass cylinder (20 cm diameter and 30 cm height) filled with warm water (25 °C). Mice were placed in the cylinder, and their swimming behavior was recorded using a camcorder for 6 min. The immobility time in the last 5 min was scored manually.

#### Open field test

Mice were placed in the center of an open-field chamber (40 cm × 40 cm), and their behavior was recorded for 10 min. The recordings were analyzed automatically using Ethovision XT14 video tracking software (Noldus). The time spent in the center (center of the arena, 20 cm × 20 cm) and the total distance explored were measured.

#### Elevated plus maze

The elevated plus maze consisted of four arms elevated 40 cm above the floor: two enclosed arms with walls and two open arms. Mice were gently placed in the central region and allowed to explore freely for 10 min. Overall activity was recorded and analyzed using the SMART video tracking system (Panlab).

#### Vertical grid test

The vertical grid apparatus consisted of an open box measuring 10 × 56 × 6 cm, positioned vertically. The back side of the box was constructed with a wire mesh grid (0.8 × 0.8 cm), while the front side was open, and the remaining four sides were made of black plexiglass. Mice were acclimated to the vertical grid three times a day for two consecutive days. If a mouse failed to climb down within 300 s, the trial was repeated.

#### Y-maze test

The mice were placed in the center of a Y-maze (three arms, 30 cm long, 120° between arms), and their behavior was recorded for 10 min. The recordings were analyzed automatically using Ethovision XT14 video tracking software (Noldus). The zone alteration and maximum alteration were measured.

#### Fear conditioning and extinction test

The behavior chamber (Panlab, LE1168) measured 25 × 25 × 25 cm and featured grid floors. Before fear conditioning, mice were habituated to the investigator’s handling for three consecutive days. On day 1, mice were habituated to the conditioning box for 5 min, followed by five mild foot shocks (0.3 mA for 0.5 s) paired with five pure tones (85 dB, 2.8 kHz). After the last foot shock, mice remained in the behavioral chamber for an additional 60 s before being returned to their home cages. On day 2, mice were introduced to a novel extinction chamber and exposed to 20 presentations of the conditioning tone. On day 3, extinction retention was assessed using three test tones. Animal behavior was recorded using webcams, and freezing levels were automatically scored with Packwin software (Panlab).

#### Chemogenetic activation of LHb astrocytes

Clozapine-*N*-oxide (CNO) was delivered to activate Hm3Gq expressed in the LHb astrocytes of cKO-Gq mice. First, 100 mM CNO was dissolved in DMSO as stock and diluted 1:100 in PBS before application. Either 2 mg/kg of CNO or an equivalent volume of vehicle (1% DMSO in PBS) was applied intraperitoneally 30 min before each test.

#### Restraint stress

The stress protocol was conducted following the procedures of a previous study^51^. Mice were placed in a 50 ml polypropylene conical tube, which was well ventilated. The mouse’s body was compressed in the tube to immobilize its legs and tails. These devices completely controlled the mice. This control process was conducted from 10:00 for 90 min a day. After stress protocols, the stressed animals were tested by FST and TST for 6 min, and the last 5 min were analyzed. The restraint stress model was composed of mice that displayed an immobility time of more than 100 s during the behavioral test.

### Electrophysiology

#### Slice preparation

The mice were anesthetized with isoflurane and decapitated to remove the brain from the skull. The brains were immediately placed in ice-cold modified artificial cerebrospinal fluid (aCSF) solution containing (in mM) 175 sucrose, 20 NaCl, 3.5 KCl, 1.25 NaH_2_PO_4_, 26 NaHCO_3_, 1.3 MgCl_2_ and 11 mM d-(+)-glucose. The solutions were then gassed with 95% O_2_ and 5% CO_2_. Coronal slices (300 µm), including the LHb, were cut using a vibratome (VT1200S, Leica) and incubated in aCSF (composition in mM: 120 NaCl, 3.5 KCl, 1.25 NaH_2_PO_4_, 26 NaHCO_3_, 1.3 MgCl_2_, 2 CaCl_2_ and 11 d-(+)-glucose). The solution in the incubation chamber was aerated continuously at room temperature with 95% O_2_ and 5% CO_2_ gas.

#### Whole-cell recordings

Whole-cell recordings were obtained using an Axopatch 700B. For measuring astrocyte passive membrane currents, a glass capillary pipette was filled with a potassium-based internal solution containing 120 mM K-gluconate, 4 mM NaCl, 10 mM HEPES, 0.2 mM EGTA, 0.2 mM Na-GTP, 2 mM Mg-ATP and 1 mM MgCl_2_. For measuring the tonic α-amino-3-hydroxy-5-methyl-4-isoxazolepdlropionic acid receptor/*N*-methyl-d-aspartate receptor (AMPAR/NMDAR) current, an internal solution containing 100 mM Cs-gluconate, 5 mM NaCl, 10 mM HEPES, 20 mM TEA, 0.6 mM EGTA, 3 mM QX314, 0.3 mM Na-GTP and 4 mM Mg-ATP was used. For measuring the tonic GABAR-mediated Cl^−^ current, a cesium chloride-based internal solution containing 135 mM cesium chloride, 5 mM EGTA, 10 mM HEPES, 4 mM NaCl, 2 mM Mg-ATP, 0.5 mM Na-GTP and 10 mM QX314 was used. The pH of the internal solution was adjusted to 7.2 with KOH or CsOH and with osmolarity adjusted to around 290 mmol/kg by sucrose. Recordings were made under infrared differential interference contrast or with fluorescent detection, and cells that were three to four cell layers below the surface of 300-µm-thick slices were recorded at a bath temperature of 32.0 ± 1.0 °C. Cells were voltage clamped at –70 mV, and bath solutions were delivered to slices via superfusion driven by a peristaltic pump at a 2 ml/min flow rate. Pipette series resistance was monitored before and at the end of the recording session. The data were discarded if the series resistance changed by over 20%. Whole-cell currents were filtered at 2 kHz, digitized to 10 kHz and stored on a microcomputer (Clampex 10 software, Molecular Devices). All the recordings were completed within 5 h of slice preparation.

##### Extracellular field recordings

Field excitatory postsynaptic potentials (ƒEPSPs) were recorded in the LHb with aCSF-filled 4–5 MΩ glass pipettes using WinLTP software (winltp.com, The University of Bristol) and National Instruments Data Acquisition (BNC-2110). Current stimuli were delivered to a constant stimulus isolator (World Precision Instruments, Inc.) using WinLTP 2.31 software. The stimulation rate was 0.067 Hz for all ƒEPSP recording experiments. After obtaining a stable baseline for 30 min, long-term depression (LTD) was induced by applying 900 pulses at 1 Hz (low-frequency stimulation (LFS) 100 µs pulse duration) through a concentric bipolar electrode (FHC, Bowdoin; 125 µm/Rnd/25 µm Pt-lr) with the same stimulating intensity used during baseline. The response amplitude was used to assess the changes in synaptic strength.

##### Drug treatment

For drug application, the aCSF solution was switched to the drug solution at least 15 min before LFS. dl-threo-β-benzyloxyaspartate (Cat. No. 1223), (+)-5-methyl-10,11-dihydroxy-5*H*-dibenzo(*a*,*d*)cyclohepten-5,10-imine Cat. No. 0924 and 6-imino-3-(4-methoxyphenyl)-1(6*H*)-pyridazinebutanoic acid hydrobromide Cat. No. 1262 were purchased from Tocris.

#### In vivo microdialysis combined with glutamate assay

Mice were anesthetized and placed on stereotaxic apparatus, as described above. A guide cannula (CMA7, CAM Microdialysis) was implemented above the left hemisphere of the LHb (ML ±0.5 mm, AP ±1.4 mm and DV −2.9 mm from the bregma). At least 7 days after surgery, microdialysis probes were inserted into the brain and experiments were carried out in freely moving animals. Dialysis probes (CMA7, CAM Microdialysis; 0.24 mm outer diameter, with 6,000 Da molecular weight cutoff) were located 1 mm from the guide cannula. The mouse was placed in a Perspex cage and the inlet cannula was connected by polythene tubing (Portex Ltd.) to a 1 ml syringe, mounted on a CMA/102 microinjection pump (CMA Microdialysis) containing aCSF (composition in mM: 147 NaCl, 2.7 KCl, 1.2 CaCl_2_ and 0.85 MgCl_2_) with CMA perfusion fluid for the central nervous system. Probes were perfused with aCSF at a constant flow rate of 2 μl/min. Samples were automatically collected every 20 min using CMA 470 refrigerated microfraction collector and stored at −70 °C until analyzed.

##### Glutamate assay

To examine the extracellular glutamate concentration in the LHb region, we performed a glutamate assay utilizing ELISA. According to the instructions of the Glutamate Assay Kit (MAK004, Sigma-Aldrich), a glutamate standard curve was determined in a linear range from 0 to 10 nmol. Collected microdialysis samples were loaded within a 96-well plate (Eppendorf, 13-690-075), and 40 μl of sample was collected and mixed with various reagents, according to the instructions of the kit. The absorbance was detected at 450 nm on a multimicroplate reader (Spectramax iD5 multimode microplate reader, Molecular Devices). The amount of glutamate present in the sample was determined from the standard curve: *C* = *S*_a_/*S*_v_ (where *S*_a_ is the amount of glutamate in an unknown sample (nmol) from the standard curve, *S*_v_ is the sample volume (µl) added into the wells and *C* is the concentration of glutamate in a sample (glutamate molecular weight: 147.3 g/mol)).

#### Statistics

Statistical analysis was performed using GraphPad Prism 10.0 (GraphPad Software, Inc.), and the values are presented as a mean ± s.e.m. When multiple samples were taken from a single animal, the number of animals used in each dataset was represented as *N* = *, and the number of sample points was denoted as *n* = *. Comparisons between two experimental groups were assessed using a Student’s *t*-test (unpaired, two tailed) unless otherwise stated, and experiments with two or more testing variables were compared using two-way analysis of variance (ANOVA) with Sidak’s post hoc tests for multiple comparisons.

## Results

### Expression and validation of astrocytic PLCη1 in the LHb

**Fig. 1:**
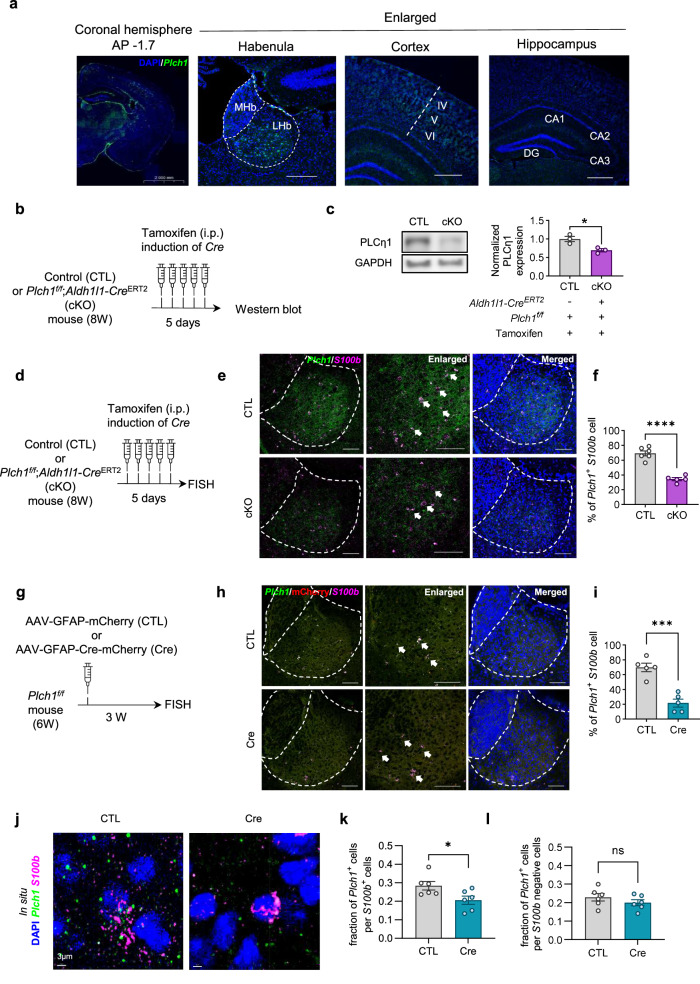
Expression of PLCη1 in mouse LHb astrocytes. **a** FISH analysis of *Plch1* mRNA on coronal mouse brain sections reveals localized expression in the thalamic and habenula regions, compared with cortical and hippocampal areas (CA1-3 hippocampal subfields, DG dentate gyrus). **b** A schematic diagram illustrating the preparation of tamoxifen-induced *Plch1* cKO or CTL mice for western blot experiment. **c** Western blot analysis of LHb tissue lysates shows a significant reduction in PLCη1 protein in *Plch1* cKO mice (CTL, 1 ± 0.072, *n* = 3; cKO, 0.694 ± 0.044, *n* = 3; *P* = 0.022; where *n* indicates the number of pooled sample vials, each containing samples from three nonoverlapping animals). **d** A schematic diagram illustrating the preparation of tamoxifen-induced *Plch1* cKO or CTL mice for western blot experiment. **e** FISH analysis in the LHb for the combination of *Plch1* with *S100b* (astrocytic marker). The dashed lines distinguish the LHb region from the medial habenula (MHb), thalamus and ventricles. Enlarged images (white boxes) show co-localized astrocyte areas indicated by smaller line boxes. **f** Quantification of *Plch1*-positive astrocytes in the LHb. The bar graph shows the percentage of *Plch1*-positive cells within the *S100b*-positive astrocytic population in CTL and *Plch1* cKO mice. *Plch1* expression was significantly reduced in the astrocytes of *Plch1* cKO mice (34.44 ± 3.38%; *n* = 6) compared with CTL mice (69.28 ± 2.06%; *n* = 5; *P* < 0.0001), indicating effective astrocyte-specific deletion of *Plch1*. Data are presented as mean ± s.e.m. *n* is the number of analyzed cells per group. **g** A schematic diagram illustrating the preparation of AAV-induced *Plch1* cKO or CTL mice for FISH experiments. **h** FISH analysis in the LHb for the combination of *Plch1* with *S100b* (astrocytic marker) in the AAV-induced cKO model. The dashed lines indicate the LHb region, and enlarged images (white boxes) highlight co-localized astrocyte areas. **i** Quantif**i**cation of *Plch1*-positive astrocytes in the LHb for the AAV-induced cKO model. The bar graph shows a significant reduction in the percentage of *Plch1*-positive cells within the *S100b*-positive astrocytic population in Cre mice (21.67 ± 5.43%; *n* = 5) compared with CTL (69.74 ± 5.71%; *n* = 5; *P* < 0.0001). **j** A 3D reconstruction of the LHb using IMARIS 9.0 software. DAPI (nuclei), *Plch1* mRNA and *S100b* (astrocytic marker) signals were detected and analyzed. **k** Quantification of the fraction of *Plch1-*positive cells among *S100b*-positive cells were significantly lower in the Cre (0.206 ± 0.022, *n* = 6) groups than CTL (0.284 ± 0.022, *n* = 6), *P* = 0.035. **l** Quantification of the fraction of *Plch1*-positive cells among *S100b*-negative cells. There was no significant differences between groups (CTL, 0.229 ± 0.020; Cre, 0.199 ± 0.016; *P* = 0.284) *P < 0.05, ***P < 0.001 and ****P < 0.0001. Data are presented as mean ± s.e.m. *n* refers to the number of animals, unless otherwise stated. Six pairs of left and right hemispheric LHb images were used for analysis for each animal.

First, we sought to determine the expression pattern of PLCη1 in the LHb. FISH analysis revealed distinct *Plch1* mRNA signals within the LHb region, suggesting a robust expression of PLCη1 in LHb compared with the expression of PLCη1 in the cortex or hippocampus (Fig. 1a).

To investigate the physiological function of PLCη1 in LHb astrocytes and further validate its expression, we developed an inducible astrocyte-specific PLCη1 conditional knockout (cKO) mouse model by breeding *Aldh1l1-Cre*^*ERT2*^ and *Plch1*^*f/f*^ mice (Fig. 1b). The inducible nature of the cKO model allows for the temporal control of gene deletion, avoiding potential developmental disturbances that might arise with a constitutive knockout.

To confirm the effectiveness of our model, we collected and pooled LHb tissues by dissecting brain slices from CTL or cKO mice. The samples were used for western blotting (Fig. 1c and see Supplementary Fig. 1 for the uncropped blot). The results demonstrate approximately 30% decreases in PLCη1 expression compared with CTL (*Plch1*^*f/f*^ mice without the *Aldh1l1-Cre*^*ERT2*^ allele).

To further validate the astrocytic expression and deletion of *Plch1*, we performed a FISH assay to assess the co-localization of *Plch1* mRNA with the astrocytic marker *S100b*. This analysis confirmed the presence of *Plch1* in *S100b*-positive astrocytes within the LHb of CTL mice, demonstrating that *Plch1* is prominently expressed in LHb astrocytes (Fig. 1d, e). To quantify the extent of *Plch1* deletion in astrocytes of *Plch1* cKO mice, we compared the fraction of *Plch1*-positive cells within the *S100b*-positive astrocytic population in the LHb. Confocal microscope images were analyzed to calculate the percentage of *S100b* cells expressing *Plch1*, and the results revealed a significant reduction of *Plch1* expression in astrocytes of *Plch1* cKO mice compared with CTL mice (Fig. 1e, f). In parallel, we evaluated whether *Plch1* expression was altered in neurons (Supplementary Fig. 2) by analyzing the expression of neuronal marker Rbfox3 (NeuN). The result indicates that there is no significant difference in the number of neurons expressing *Plch1* between CTL and cKO mice, suggesting that our cKO selectively targets astrocytes.

To complement the tamoxifen-inducible model, we used an AAV-expressing Cre recombinase under the GFAP promoter (AAV-GFAP-Cre) to selectively suppress *Plch1* in LHb astrocytes. Consistent with the tamoxifen model, this approach significantly reduced *Plch1* expression in astrocytes (Fig. 1g–i).

Using IMARIS 9.0 software, we reconstructed LHb regions in 3D and analyzed DAPI (nuclei), *Plch1* mRNA and *S100b* (astrocyte marker) signals (Fig. 1j). Approximately 200 cells per sample were detected (CTL: 173 ± 10.34; Cre: 213.11 ± 6.63), with ~15% of the cells identified as *S100b*-positive astrocytes (CTL: 27.08 ± 1.65; Cre: 28.56 ± 2.15). Samples were derived from six animals per group, with three slices per animal and bilateral hemispheric analysis. Importantly, the fraction of *Plch1*-positive cells among *S100b*-positive astrocytes was significantly lower in the Cre group, while non-astrocytic (*S100b*-negative) cells showed no change in *Plch1* expression. (Fig. 1k, l) These findings confirm the astrocyte-specific suppression of *Plch1* in the viral model.

### Alterations in morphological complexities and electrophysiological traits of LHb astrocytes in *Plch1* cKO mice

**Fig. 2:**
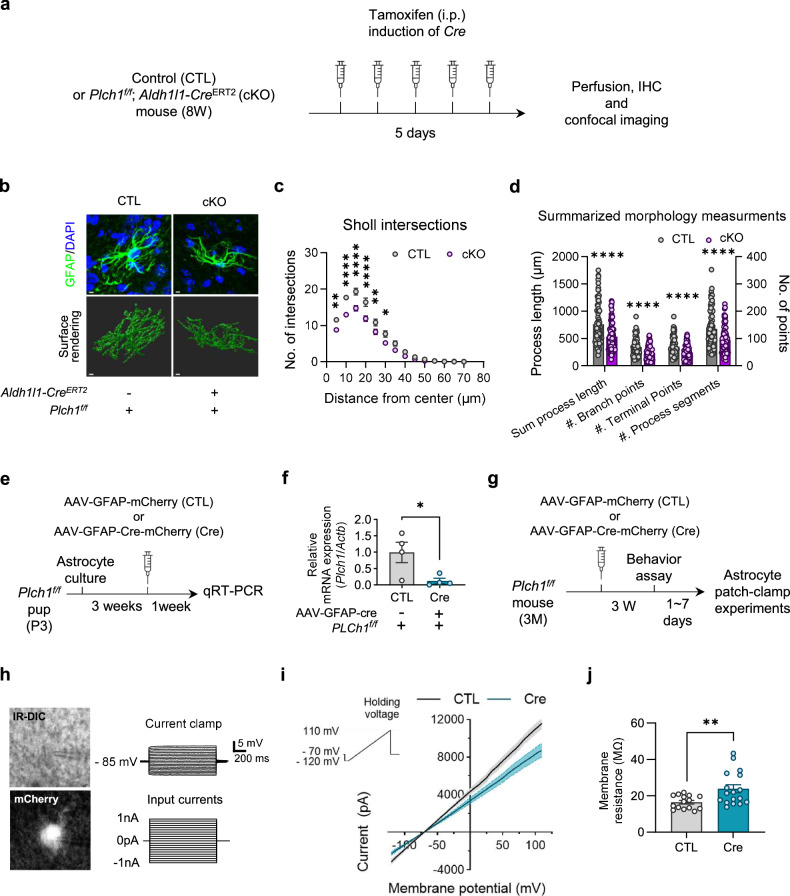
Deletion of PLCη1 leads to structural and functional changes in LHb astrocytes. **a** A schematic diagram of tamoxifen-induced cKO or CTL mice. **b** Representative confocal images and surface renderings of 3D reconstruction. Scale bar, 5 µm. **c** A Sholl intersection plot showing intersections within a 5–70 µm radius from the center of GFAP filament in the LHb (CTL: *n* = 87, *N* = 7; cKO: *n* = 88, *N* = 7; two-way ANOVA interaction effect, *P* = 0.006; distance from the center effect, *P* < 0.0001; group effect, *P* < 0.0001; post hoc multiple comparison, *P* = 0.003 for 5 µm, *P* < 0.0001 for 10–20 µm, *P* = 0.068 for 25 µm and *P* = 0.010 for 90 µm. *n* indicates the number of cells and *N* refers to the total animals). **d** Detailed measurement results demonstrating that astrocyte process complexity is significantly reduced in *Plch1* cKO mice (total process length: CTL, 760.966 ± 39.756 µm; cKO, 532.648 ± 27.388 µm, *P* < 0.0001; number of branch points: CTL, 67.943 ± 3.543; cKO, 47.318 ± 2.570, *P* < 0.0001; number of terminal points: CTL, 69.862 ± 3.672; cKO, 48.670 ± 2.633, *P* < 0.0001; number of process segments: CTL, 135.552 ± 7.224; cKO, 94.091 ± 5.172, *P* < 0.0001). **e** A schematic illustration of the cultured astrocytes used for estimation of viral efficacy of deleting *Plch1* in *Plch1*^*f/f*^ cells. **f** qRT–PCR shows a significant reduction of PLCη1 mRNA (CTL, 0.993 ± 0.311, *n* = 4; Cre, 0.120 ± 0.081, n = 4, *P* = 0.035; *n* refers a cultured cells from each independent animal). **g** A schematic illustration of CTL and Cre mice used in the astrocyte patch-clamp experiment. **h** Identification of LHb astrocytes were carried by fluorescence visualization of mCherry signal before whole-cell patch and confirming the absence of action potential against positive current injection in current-clamp during whole-cell patch. **i** The voltage ramp (the voltage command is depicted in the inset) protocol was applied to cell and the I–V relationship was established. **j** The LHb astrocyte membrane resistances calculated from the I–V curve were significantly bigger in Cre mice (CTL, 16.472 ± 0.953, *n* = 15, *N* = 4; Cre, 23.807 ± 2.230, *n* = 17, *N* = 4, *P* = 0.0072). **P < 0.001. Data are presented as mean ± s.e.m.

Recent evidence suggested that PLCη2 regulates neuronal dendrite outgrowth^23^, therefore we questioned whether astrocytic PLCη1 has a similar function for cellular morphology. We conducted an IHC experiment, staining astrocyte processes in the LHb of brain cryosection obtained from tamoxifen-injected CTL and cKO mice (Fig. 2a). We imaged the stained cryosections with a high-resolution confocal microscope. The surface renderings were created with Imaris 9.0 software (Fig. 2b). Detailed analysis using filament modules (Fig. 2c, d) showed that cKO led to a noticeable morphological alteration in astrocytes. We found that Sholl intersections (the number of intersections with concentric circles at increasing distances from the processing center) at a radius from 5 µm to 30 µm were significantly reduced in astrocytes of cKO mice. Additionally, we observed an approximately 20% reduction in the total process length, number of branch points, terminal points and process segments. These results suggest that the deletion of PLCη1 leads to attenuated outgrowth of the astrocyte process and reduced morphological complexity in LHb.

To further investigate the functional consequences of *Plch1* deletion in LHb astrocytes, we switched from the tamoxifen-inducible cKO mouse model to a viral approach using an AAV-expressing Cre recombinase under the control of the GFAP promoter (AAV-GFAP-Cre). This allowed us to visually distinguish astrocytes from other cell types during electrophysiological assessments by co-expressing mCherry under the GFAP promoter (AAV-GFAP-mCherry) (Fig. 2e).

Before whole-cell patch-clamp experiments, the efficacy of AAV-GFAP-Cre transduction was confirmed by conducting qRT–PCR analysis (Fig. 2e, f). CTL or AAV-GFAP-Cre (Cre) virus was transfected into primary astrocyte cultures obtained from *Plch1*^*f/f*^ pups. *Plch1* mRNA levels in Cre mice were significantly lower than in CTL mice, demonstrating the ability of the virus to attenuate *Plch1* mRNA expression in *Plch1*^*f/f*^ astrocytes.

Then, we measured membrane conductance in LHb astrocytes via whole-cell patch-clamp experiments (Fig. 2f, g). Astrocytes were identified from other brain cells by their GFAP-dependent reporter mCherry signal (Fig. 2h). Furthermore, the identification was confirmed after whole-cell configuration, with the absence of action potential during positive current injections up to 1 nA confirming their identity.

A depolarizing ramp protocol (from −120 to 110 mV) was applied in voltage-clamp mode to evaluate the passive membrane properties of the astrocytes and the current-voltage (*I*–*V*) relationship was established (Fig. 2i), revealing a decrease in membrane conductance in astrocytes from Cre mice. The membrane resistance, calculated from linear fits of the *I*–*V* curve, was significantly higher in the Cre (Fig. 2j). This elevation in membrane resistance is possibly associated with the reduction of morphological complexities and potential result of decreased surface channel expression in PLCη1-deleted astrocytes (Fig. 2c, d).

Our focus on the functional implications of the PLCη1 deletion in astrocytes led us to study intracellular Ca^2+^ signaling, given the known role of PLC in governing these processes. A ratiometric calcium imaging experiment was conducted in cultured astrocytes, and we found that the Ca^2+^ response to bradykinin treatment was significantly attenuated when PLCη1 was silenced via short hairpin RNA delivered through a virus vector (Supplementary Fig. 3). Considering that gliotransmission can be regulated by bradykinin through inducing astrocytic Ca^2+^ activity^52^, this result suggests that alterations of the PLCη1 availability in astrocytes affect not only their morphological and electrophysiological characteristics, but also their calcium-signaling pathways, which can potentially influence broader neurophysiological functions.

### Deletion of astrocytic PLCη1 enhances SM–LHb synapse and impairs impairs extrasynaptic LTD

**Fig. 3:**
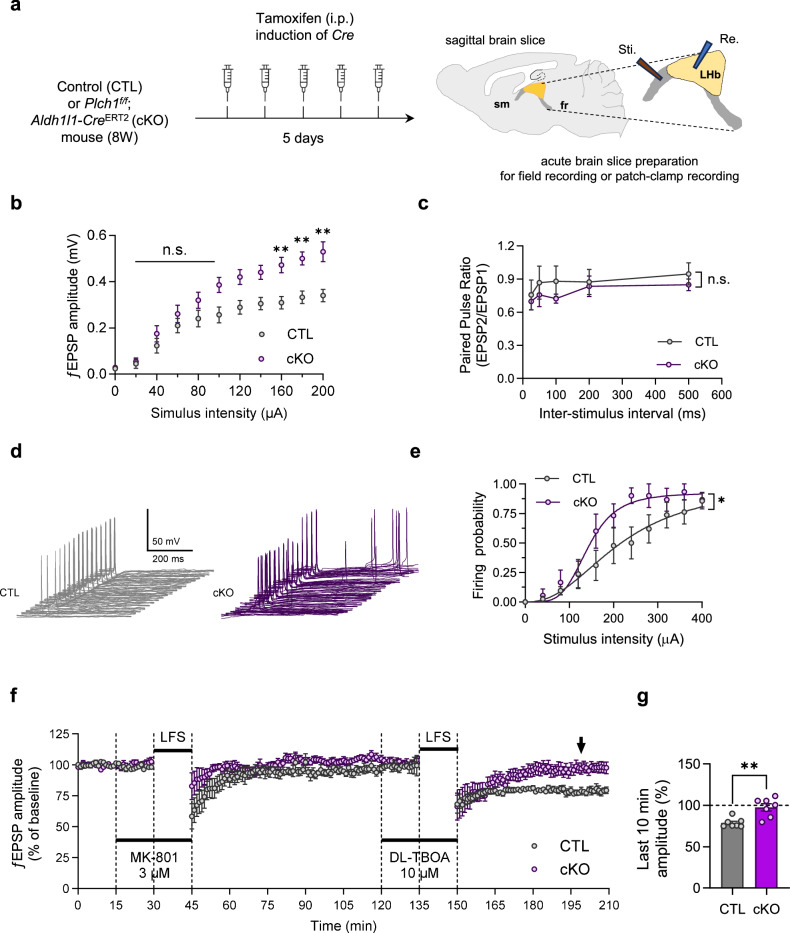
Deletion of astrocytic PLCη1 increases LHb synaptic efficacy and response firing probability in *Plch1* cKO mice. **a** The experimental scheme illustrates the preparation of the CTL and *Plch1* cKO mice. **b**, **c** Assessment of basal transmission at the SM–LHb synapse in CTL and *Plch1* cKO mice: an input–output relationship measurement between the current intensity (0–200 μA) and evoked ƒEPSP amplitude (mV) shows enhanced synaptic efficacy in cKO mice (CTL, *n* = 4, *N* = 4; cKO, *n* = 7, *N* = 7; *n* indicates number of brain tissue and *N* refers to the total animals; two-way ANOVA, group factor, *P* < 0.0001, post hoc multiple comparison, *P* = 0.0100 for 80 μA, 0.0068 for 90 μA and 0.0013 for 100 μA) (**b**) and the paired pulse ratio of ƒEPSP amplitudes across varied interstimulus intervals (25, 50, 100, 200 and 500 ms) shows no significant difference between CTL and *Plch1* cKO mice (CTL, *n* = 4, *N* = 4; cKO, *n* = 5, *N* = 5; *n* and *N* as in **b**) (**c**). **d** Superimposed traces of firing EPSPs evoked by varying (20–180 µA) SM stimulation are shown. **e** The mean firing probability for each stimulus strength is shown. The sigmoidal fits to each dataset exhibited statistically significant differences. IC_50_ of CTL (216.891 ± 77.956 µA, 95% confidence interval, *n* = 7, *N* = 5), was higher than cKO (142.424 ± 10.112 µA, 95% confidence interval, *n* = 6, *N* = 5; *n* refers the number of cells measured and *N* refers the total number of animals). **f** After masking synaptic NMDAR by MK-801, DL-TBOA treatment combined with LFS induced an extrasynaptic LTD in the CTL mice but did not induced in *Plch1* cKO mice. Time courses of the ƒEPSP amplitude are displayed. **g** The means ƒEPSP amplitude of the last 10 min (arrow) were compared (CTL, 78.739 ± 2.102%, *n* = 7, *N* = 7; cKO, 97.508 ± 4.319%, *n* = 7, *N* = 7, *P* = 0.002; *n* refers the number of slices recorded and *N* refers the total number of animals). **P < 0.05 and **P < 0.01. Data are presented as mean ± s.e.m.

With afferent stria medullaris (SM) and efferent fasciculus retroflexus, the habenula forms a crucial part of the diencephalic conduction system. For a deeper understanding of how astrocytic alterations might influence this transmission, we investigated potential changes in the SM by recording evoked ƒEPSP in sagittal LHb slices of CTL and cKO mice (Fig. 3a). We measured the input–output relationship across a range of stimulus intensities from 0 to 200 μA (Fig. 3b). Our results revealed significantly larger amplitudes of evoked ƒEPSPs in cKO mice compared with CTL mice, indicating enhanced synaptic strength in the LHb of cKO mice.

To determine whether presynaptic mechanisms contributed to this enhanced synaptic response, we assessed the paired-pulse ratio at various interstimulus intervals (25, 50, 100, 200 and 500 ms). However, no significant differences in the paired-pulse ratio (EPSP2/EPSP1) were observed in the cKO group (two-way ANOVA, group factor *P* = 0.158; Fig. 3c). These findings suggest that the potentiated synaptic strength may arise from postsynaptic changes.

Next, we examined whether changes in synaptic efficacy affected the output firing responses of LHb neurons. Using a current-clamp configuration, we performed electrophysiological recordings to estimate the probability of evoked action potentials in response to electrical stimulation of the SM across various intensities (0–400 µA in 40 µA intervals; Fig. 3d, e). The results were fit to a sigmoidal model with two constraints: the top of the fit curve was restricted to less than 1 and the bottom was fixed at 0. Statistical analysis revealed that the two sigmoidal fits were significantly different (*F*_2,127_ = 3.962, *P* = 0.021, extra sum of four squares test) and the IC_50_ of the *Plch1* cKO mice was lower than that of the CTL, suggesting that LHb neurons in the cKO mice exhibit an increased propensity to fire in response to synaptic inputs. These results indicate that the deletion of astrocytic PLCη1 enhances basal synaptic transmission and increases the response firing probability in the cKO group.

Next we aimed to investigate how astrocytic alterations in *Plch1* cKO mice might influence synaptic function, particularly in the context of gliotransmission, which heavily depends on calcium-signaling pathways^17,21,39,53,54^. Given our observation of reduced intracellular calcium responses in *Plch1* cKO astrocytes, it is plausible that the deletion of astrocytic PLCη1 disrupts calcium-dependent gliotransmission, a mechanism known to modulate neuronal activity via extrasynaptic receptors^55^. Additionally, our previous findings demonstrated increased synaptic connectivity in the LHb of *Plch1* cKO mice, prompting us to explore the functional consequences on extrasynaptic plasticity.

To this end, we investigated whether extrasynaptic LTD—a form of plasticity previously reported in the LHb in an earlier study^56^—remains intact in the brain slice of cKO mice (Fig. 3f, g). To isolate extrasynaptic NMDA receptor contributions and minimize synaptic NMDAR effects on plasticity, we used the activity-dependent NMDA receptor antagonist MK-801 (3 μM)^56,57^. Following a 30 min baseline stabilization, MK-801 was applied to the recording chamber for 15 min to block synaptic NMDAR activity. Next, we tested a well-known NMDAR-dependent form of neuroplasticity, synaptic LTD, using LFS (1 Hz, 900 pulse) and LFS failed to induce synaptic LTD in both CTL and *Plch1* cKO mice (Fig. 3f, from 0 to 120 min). To preferentially activate extrasynaptic NMDA receptors, we inhibited glutamate transporter GLT-1 with dl-TBOA (10 μM), which increases glutamate spillover from synaptic clefts. dl-TBOA treatment, combined with another train of LFS, successfully induced extrasynaptic LTD in LHb slices from CTL mice. However, this extrasynaptic LTD was absent in slices from *Plch1* cKO mice (Fig. 3f, from 120 to 150 min). A comparison of normalized ƒEPSP amplitudes during the last 10 min of recording revealed significantly lower amplitudes in CTL slices compared with *Plch1* cKO slices (Fig. 3g), indicating that deletion of astrocytic PLCη1 impairs extrasynaptic LTD.

### Deletion of astrocytic PLCη1 reduces tonic AMPAR/NMDAR current and in the LHb and a chemogenetic activation of astrocytes rescues the current

**Fig. 4:**
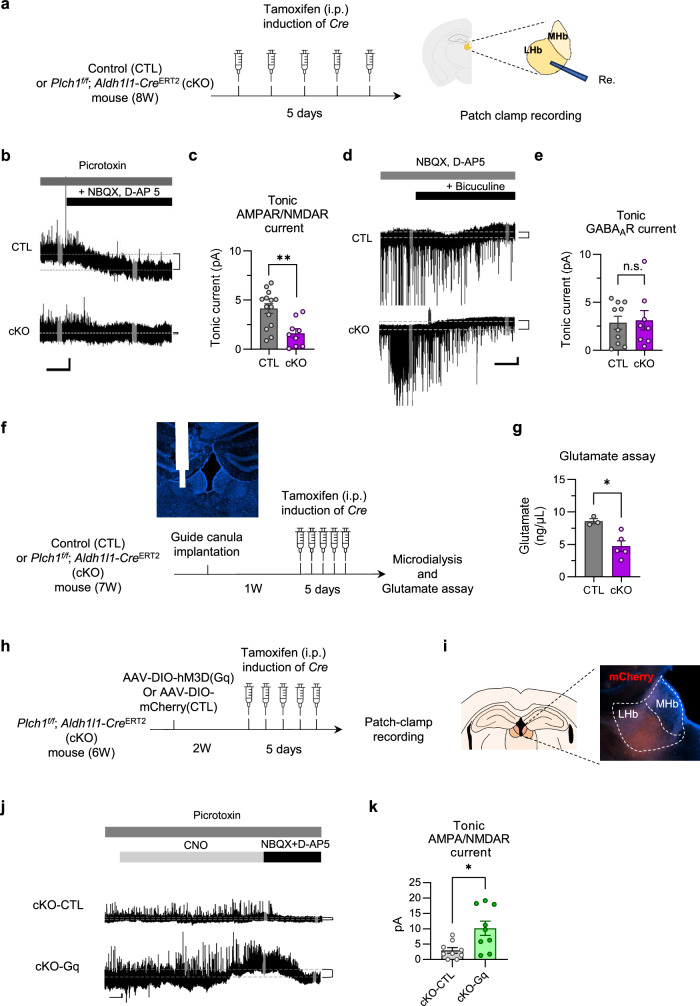
Deletion of astrocytic PLCη1 reduces tonic AMPAR/NMDAR current and impairs extrasynaptic LTD in the LHb. **a** The experimental scheme depicting the preparation of CTL and *Plch1* cKO mice and brain slices for electrophysiology. Tonic currents were estimated on a coronal brain slice and extrasynaptic LTD was evaluated on a sagittal brain slice. **b** Representative traces show attenuated tonic AMPAR/NMDAR in the *Plch1* cKO mice. Each 10 s portion (gray parts of traces) was compared before and after the drug treatment (dashed gray lines and brackets between the lines). Scale bars, 50 s and 5 pA. **c** The summarized mean tonic AMPAR/NMDAR current plot shows a significant difference between CLT and *Plch1* cKO mice (CTL, 4.141 ± 0.497 pA, *n* = 14, *N* = 8; cKO, 1.622 ± 0.453 pA; *n* = 9, *N* = 6; *P* = 0.002; *n* refers the number of cells measured and *N* refers the total number of animals). **d** Representative traces show that the magnitude of tonic GABA_A_R current is not different in CTL and *Plch1* cKO mice. **e** The summarized plot of tonic GABA_A_R current shows no significant difference between CLT and *Plch1* cKO mice (CTL, 2.867 ± 0.676 pA, *n* = 10, *N* = 6; cKO, 3.126 ± 1.024 pA, *n* = 8, *N* = 6; *P* = 0.830; *n* and *N* as in **c**). **f** A schematic representation of the microdialysis. **g** Quantification of extracellular glutamate levels in the LHb. Microdialysis followed by glutamate assay revealed a significant reduction in extracellular glutamate in *Plch1* cKO mice compared with CTL mice (CTL, 8.578 ± 0.375 ng/μl, *n* = 3; cKO, 4.724 ± 0.831 ng/μl, *n* = 5, *P* = 0.0015; *n* refers the number of samples and a sample is derived from an animal). **h** A schematic representation of the CNO-inducible astrocytes in cKO mouse LHb. **i** mCherry expression of viral infection was confirmed after behavioral tests. **j** Representative traces of tonic AMPAR/NMDAR currents of both groups. Tonic currents were restored in *Plch1* cKO mice following CNO application in cKO-Gq but not in cKO-CTL. Scale bars, 50 s and 5 pA. **k** Quantification of tonic AMPAR/NMDAR currents. With 10 min of CNO pretreatment, cKO-Gq mice showed significantly more tonic AMPAR/NMDAR current than cKO-CTL mice (cKO-CTL, 2.944 ± 0.907 pA, *n* = 9, *N* = 5; cKO-Gq, 10.14 ± 2.33 pA, *n* = 9, *N* = 5, *P* = 0.011; *n* refers the number of cells recorded and *N* refers the number of animal). *P < 0.05 and **P < 0.01. Data are presented as mean ± s.e.m.

Our result suggests potentially insufficient extrasynaptic glutamate supplies in the cKO mice. To test whether the deletion of astrocytic PLCη1 impacted gliotransmission, we decided to estimate tonic AMPAR/NMDAR and γ-aminobutyric acid subtype A receptor (GABA_A_R) currents in LHb from CTL or cKO mice (Fig. 4a–e). These measurements aimed to evaluate whether astrocytic PLCη1 deletion affects the gliotransmitter signaling in the LHb. Tonic AMPAR/NMDAR currents were identified by comparing the baseline membrane currents at a clamped voltage of +40 mV during the application of a GABA receptor channel blocker (picrotoxin), and the currents after the addition of glutamate blockers (NBQX for AMPA receptors and D-AP5 for NMDA receptors) (Fig. 4b, c). We found that cKO mice showed significantly smaller tonic AMPAR/NMDAR currents than CTL mice, suggesting decreased tonic excitation in LHb neurons. Similarly, we measured the tonic GABA_A_R currents (Fig. 4d, e) by comparing the baseline currents before and after bicuculline (an ionotropic GABA_A_ channel-specific blocker) treatment with ambient blockade of glutamatergic channels. Unlike AMPAR/NMDAR currents, we found no significant (*P* = 0.945) difference in GABA_A_R currents between CTL and *Plch1* cKO mice.

To investigate whether the observed changes in tonic current were linked to a shortage of extrasynaptic glutamate, we performed microdialysis experiments. Guide cannulas were implanted into the left LHb hemisphere of CTL and *Plch1* cKO mice (Fig. 4e). Following tamoxifen-induced *Plch1* deletion, extracellular fluid from the LHb was collected via microdialysis and assayed to quantify glutamate levels (Fig. 4f). The results revealed a significant reduction in extracellular glutamate in *Plch1* cKO mice compared with CTL mice (Fig. 4g). These findings suggest that astrocytic PLCη1 deletion reduces extrasynaptic glutamate availability, potentially contributing to the observed deficits in tonic AMPAR/NMDAR currents.

To further confirm the role of astrocytic PLCη1 in regulating tonic glutamate signaling, we utilized a chemogenetic approach to activate LHb astrocytes (Fig. 4h, i). The LHb of CTL and *Plch1* cKO mice was infected with an AAV encoding hM3D(Gq), a Gq-coupled designer receptor activated exclusively by designer drugs, driven by Cre recombinase. Viral expression was verified on postfixed brain slices via fluorescent imaging.

We performed patch-clamp recordings in LHb slices. Bath application of 10 μM CNO for 10 min to activate hM3Dq-expressing astrocytes significantly restored the reduced tonic AMPAR/NMDAR currents in *Plch1* cKO mice (Fig. 4j, k). These findings demonstrate that restoring astrocytic calcium signaling via chemogenetic activation rescues tonic glutamate signaling, further implicating astrocytic PLCη1 in the regulation of gliotransmitter release.

### Deletion of astrocytic PLCη1 in LHb induces depressive-like behaviors and a chemogenetic activation of astrocytes rescues the changes

**Fig. 5:**
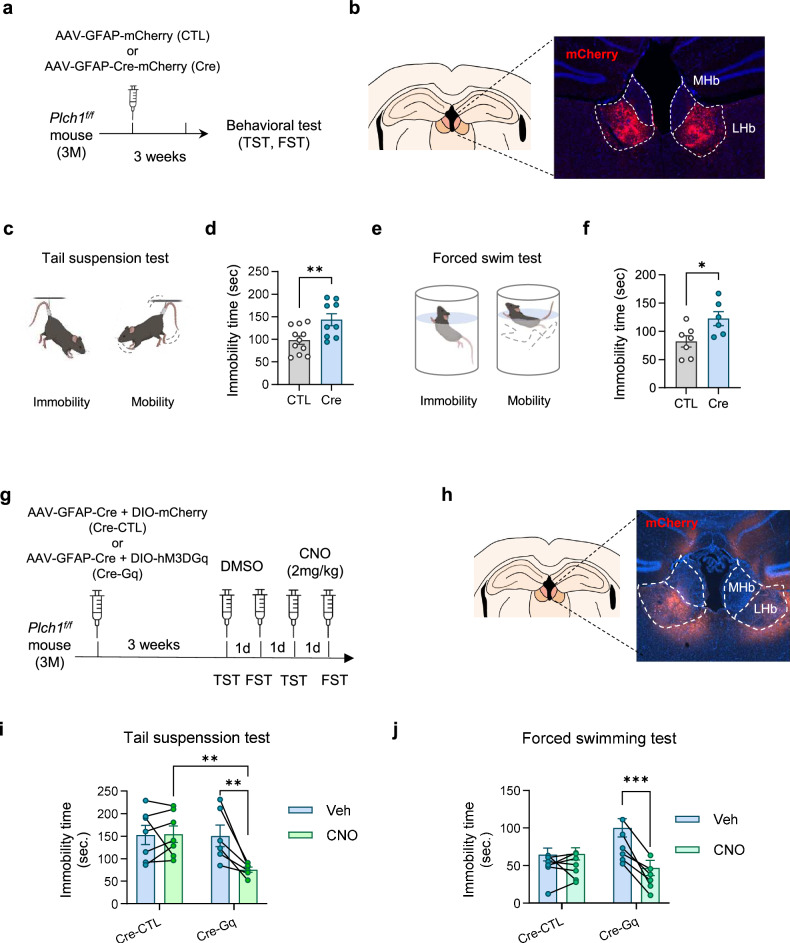
Deletion of astrocytic PLCη1 in the LHb affects depressive-like behavior. **a** The experimental scheme depicts the preparation of CTL and Cre virus-injected mice for behavioral assessments. **b** The injection sites were confirmed to be in the LHb after behavioral assays to exclude any off-target subjects from analysis. **c** A schematic representation of the mobile and immobile states of mice during the FST. **d** Bar graphs displaying the mean of immobility duration for CTL and Cre mice (CTL, 82.28 ± 10.06 s, *N* = 7; Cre 122.66 ± 12.35 s, *N* = 6, *P* = 0.02). **e** A schematic representation of the mobile and immobile states of mice during the TST. **f** Bar graphs displaying the mean of immobility duration for CTL and Cre mice (CTL, 82.28 ± 10.06 s, *N* = 7; Cre 122.66 ± 12.35 s, *N* = 6, *P* = 0.027). **g** A schematic representation of the chemogenetic experiment. *Plch1*^*f/f*^mice were injected with AAV-GFAP-Cre combined with either DIO-mCherry (cKO-CTL) or DIO-hM3D(Gq) (cKO-Gq) to knock down *Plch1* and induce astrocyte-specific expression of the DIO constructs in the LHb. **h** Fluorescence imaging following the behavioral experiments conducted to confirm viral infection in the LHb. **i** During the TST, the immobility time is significantly reduced in the CNO session of cKO-Gq compared with cKO-CTL and Veh session of cKO-Gq (Cre-CTL, Veh, 152.57 ± 21.12 s; Cre-CTL, CNO, 154.36 ± 18.49; Cre-Gq, Veh, 150.43 ± 23.88 s; Cre-Gq, CNO, 75.63 ± 5.66 s), No significant differences were observed in the cKO-CTL group between vehicle and CNO treatment. **j** The immobility time during FST is significantly reduced in the CNO session of cKO-Gq compared with the Veh session cKO-Gq, while there was no difference across sessions in cKO-Cre mice (Cre-CTL, Veh, 64.71 ± 8.61 s; Cre-CTL, CNO, 65.63 ± 8.09 s; Cre-Gq, Veh, 100.29 ± 12.1 9 s; Cre-Gq, CNO, 46.79 ± 10.11 s). *N* = 7 and 6 for Cre-CTL and Cre-Gq, respectively. *P < 0.05, **P < 0.01, ***P < 0.001 and ****P < 0.0001. *N* refers to the total number of animals. Data are presented as mean ± s.e.m.

The neuronal changes observed in the LHb of *Plch1* cKO mice prompted us to investigate their impact on mood regulation. To restrict the temporal and spatial influence of astrocytic PLCη1 deletion, we employed targeted viral injections of Cre recombinase under a GFAP promoter (Fig. 5a, b). This allowed us to selectively delete astrocytic *Plch1* in the LHb and establish a clear relationship between LHb astrocytic PLCη1 deletion and depressive-like behaviors. AAV-GFAP-Cre or CTL virus was injected into the LHb of *Plch1*^*f/f*^ mice 2–3 weeks before behavioral testing. Injection sites were confirmed to avoid off-target effects (Fig. 5b and Supplementary Fig. 4).

We first assessed depressive-like behaviors using the FST (Fig. 5c). During the 5 min analysis period, Cre-injected mice displayed a significantly increased immobility time compared with CTLs (*P* = 0.027; Fig. 5d), a behavior commonly associated with helplessness in rodents. Consistent results were observed in the TST (Fig. 5e), where Cre-injected mice also exhibited significantly increased immobility (*P* = 0.0094; Fig. 5f). To further evaluate depression-related anhedonia, we performed a sucrose preference test (Supplementary Fig. 5). Cre mice demonstrated a significantly reduced preference for the sucrose solution on day 2 (*P* = 0.046; Supplementary Fig. 5c), further supporting the development of depressive-like behaviors.

To determine whether chemogenetic activation of LHb astrocytes could rescue depressive-like behaviors in *Plch1* cKO mice, we injected *Plch1*^*f/f*^ mice with AAV-GFAP-Cre in combination of either DIO-mCherry (CTL, cKO-CTL) or DIO-hM3D(Gq) (cKO-Gq). AAV-GFAP-Cre both knocked down *Plch1* by excising the floxed allele and induced astrocyte-specific expression of the DIO construct, enabling targeted gene deletion and chemogenetic manipulation in LHb astrocytes (Fig. 5g). The viral expression was confirmed via fluorescence imaging after behavioral experiments (Fig. 5h) conducted over 4 days. Vehicle (Veh, DMSO) was administered intraperitoneally on days 1 and 2, followed by 2 mg/kg CNO i.p. 30 min before testing on days 3 and 4. Testing alternated between the TST and FST: day 1 (Veh–TST), day 2 (Veh–FST), day 3 (CNO–TST) and day 4 (CNO–FST).

Two-way ANOVA revealed a significant interaction between treatment (DMSO versus CNO) and group (cKO-CTL versus cKO-Gq; Fig. 5i, j). CNO treatment to cKO-Gq mice lead to significantly less immobility in both TST and FST compared with vehicle-treated session (post hoc test, *P* = 0.0022 for TST and *P* = 0.0003 for FST), whereas mCherry CTLs showed no differences. These results indicate that chemogenetic activation of LHb astrocytes restores depressive-like behaviors in *Plch1* cKO mice.

We assessed anxiety-like behavior using the open-field test and elevated plus maze test and evaluated motor functionality through the vertical grid test (Supplementary Fig. 6). No significant differences were observed between cKO and CTL mice in total distance moved, dwell time in the center or periphery of the open field (Supplementary Fig. 6b,c), or dwell time in or entries into the open arms of the elevated plus maze (Supplementary Fig. 6e, f). Similarly, motor performance on the vertical grid test remained unaffected, as indicated by no differences in the rate of successful limb movements or the time required to climb up and down (Supplementary Fig. 6h–j). These results suggest that the deletion of astrocytic PLCη1 in the LHb does not impact anxiety-like behavior or motor function, ruling out potential confounding effects of anxiety or motor deficits in our behavioral assessments of depressive-like behaviors.

Additionally, we used the Y-maze test to investigate working memory and fear conditioning and extinction tests to study fear memory in cKO mice (Supplementary Fig. 7). We found no significant differences between the cKO and CTL mice in these cognitive functions. In addition, a sociability test revealed no differences in social behavior or motivation between the groups. These results indicated that *Plch1* knockout in LHb astrocytes did not alter working memory, fear memory or social behavior.

Our domain-specific behavioral tests show that astrocytic deletion of *Plch1* in LHb affects mood regulations while not changing motor, anxiety or cognitive functions.

### RST reduces *Plch1* expression in the LHb

**Fig. 6:**
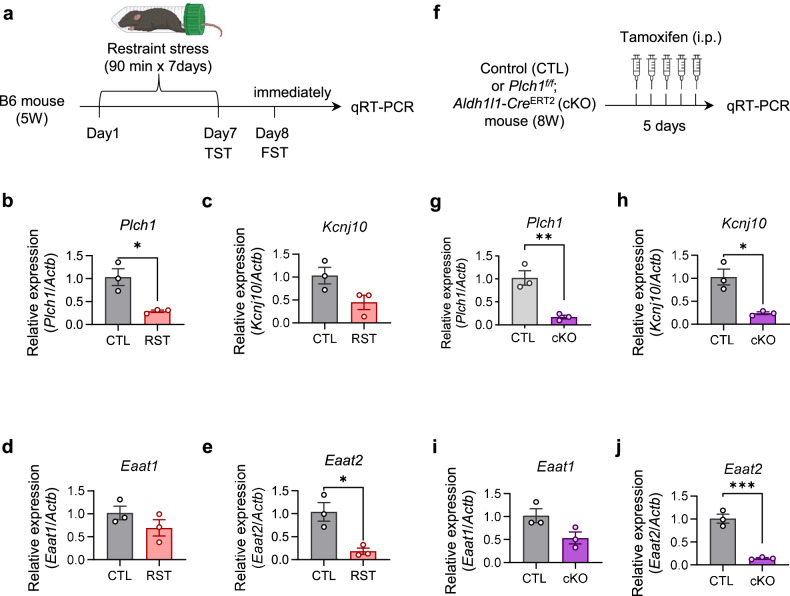
Acute stress reduces PLCη1, Kir4.1, GLAST and GLT-1 mRNA expression in the LHb of wild-type mice and astrocytic *Plch1* cKO mimics the mRNA expression patterns. **a** Schematic representation of the evaluation of mRNA expression changes in LHb following restraint stress. **b**–**e** Relative mRNA expression levels of *Plch1* (PLCη1), *Kcnj10* (Kir4.1), *Eaat1* (GLAST) and *Eaat2* (GLT-1) in LHb from CTL and RST mice, as assessed by qRT–PCR: *Plch1* (CTL, 1.030 ± 0.183 *n* = 3; RST, 0.290 ± 0.025, *P* = 0.016) expression was significantly reduced in RST mice compared with CTL (**b**); there were no significant changes in *Kcnj10* (CTL, 1.033 ± 0.182, *n* = 3; RST, 0.450 ± 0.156, *n* = 3, *P* = 0.717) (**c**) or *Eaat1* (CTL, 1.020 ± 0.149, *n* = 3; RST, 0.695 ± 0.178, *n* = 3, *P* = 0.232) (**d**) and *Eaat2* (CTL, 1.040 ± 0.203, *n* = 3; RST, 0.185 ± 0.068, *n* = 3, *P* = 0.016) also exhibited significant decreases in the RST mice relative to CTL (**e**). **f** Schematic representation of the mRNA espression changes in LHb of CTL and *Plch1* cKO mice. **g**–**j** Analogous mRNA expression levels from *Plch1* cKO mice compared with CTL: similar to the RST model, *Plch1* cKO mice demonstrated substantial downregulation of *Plch1* mRNA (CTL, 1.023 ± 0.155, *n* = 3; cKO, 0.170 ± 0.040, *n* = 3; *P* = 0.025) (**g**) and *Kcnj10* (CTL, 1.027 ± 0.175, *n* = 3; cKO, 0.240 ± 0.029, *n* = 3) (**h**); *Eaat1* (CTL, 1.020 ± 0.150, *n* = 3; 0.533 ± 0.128, *n* = 3, *P* = 0.071) (**i**) did not show significant changes and *Eaat2* (CTL, 1.010 ± 0.099, *n* = 3; cKO, 0.143 ± 0.015, *n* = 3) expression was significantly reduced (**j**). *P < 0.05, **P < 0.01 and ****P < 0.0001. *n* indicates the number of vials and three nonoverlapping animals were pooled in each vial. Data are presented as mean ± s.e.m.

As demonstrated earlier in this study, astrocytic deletion of PLCη1 led to profound changes in LHb astrocyte morphology, electrophysiology, neuronal membrane currents, synaptic properties and depressive-like behaviors. Building on these findings, we investigated the changes in *Plch1* expression in a well-established depressive-like animal model, chronic restraint stress (RST). Using this model, we probed the changes in crucial LHb astrocyte molecules using qRT–PCR (Fig. 6a–e). Alongside *Plch1*, we evaluated mRNA levels of the potassium channel Kir4.1 (*Kcnj10*) and the glutamate transporters GLAST1 (*Eaat1*) and GLT-1(*Eaat2*), which are implicated in the dysregulation of *Kir4.1*-mediated potassium buffering or *GLT-1*-dependent glutamate uptake in LHb astrocytes, which contributes to neuronal hyperexcitability and depressive-like behaviors^45,58^.

LHb tissue was obtained from mice subjected to a chronic RST model, where animals were restrained for 90 min daily from day 1 to day 7. On day 7, the mice underwent the TST, followed by the FST on day 8. RST mice exhibited profound depressive-like behaviors, characterized by significantly increased immobility times during both the TST and FST compared with CTL mice (Supplementary Fig. 8). Immediately after the FST, mice were killed to collect brain slices for RNA extraction and subsequent qRT–PCR analysis (Fig. 6a). Purified RNAs were amplified with a set of probes detecting *Plch1*, *Kcnj10*, *Eaat1* and *Eaat2*. Strikingly, *Plch1* mRNA expression was significantly lower in RST mice compared with the CTL mice (Fig. 6b). While *Kcnj10* and *Eaat1* mRNAs did not showed significant changes (Fig. 6c, d), *Eaat2* expression also declined in RST mice compared with CTL (Fig. 6e). Subsequent qRT–PCR of the same set of mRNAs from LHb tissue of tamoxifen-induced cKO mice (Fig. 6f) revealed a similar pattern of alterations in gene expression (Fig. 6g–j). Astrocytic *Plch1* knockout resulted in a reduction of *Plch1* (*P* = 0.006)and significant downregulation of *Kcnj10* and *Eaat2* (*P* = 0.042 and *P* = 0.011, respectively), whereas *Eaat1* displayed a modest, nonsignificant decrease (*P* = 0.071).

These observations in RST mice reflect the molecular alterations in astrocyte-specific *Plch1* cKO mice. This correlation emphasizes the role of PLCη1 in the LHb, consistent with its proposed involvement in mood regulation.

## Discussion

In this study, we identified *Plch1* expression in LHb astrocytes and demonstrated that astrocyte-specific *Plch1* deletion induces significant changes in astrocyte morphology, passive conductance and calcium signaling. These alterations disrupted astrocyte–neuron interactions, leading to increased synaptic efficacy, impaired extrasynaptic neuroplasticity and depressive-like behaviors in mice. Specifically, we observed reduced tonic AMPAR/NMDAR currents and extracellular glutamate levels in *Plch1* cKO mice, suggesting that *Plch1* deletion impairs gliotransmission. Chemogenetic activation of LHb astrocytes via hM3D(Gq) restored tonic currents and rescued depressive-like behaviors, highlighting the critical role of astrocytic PLCη1 in neuronal and mood regulation. Additionally, chronic restraint stress reduced the expression of *Plch1* in the LHb, emphasizing the link between astrocytic PLCη1 dysfunction and depressive-like behaviors.

Given previous evidence linking increased LHb activity to depressive-like behavior^30,45,59,60^, our findings suggest that the astrocyte-specific reduction of PLCη1 contributes significantly to this neuropsychiatric phenomenon. Notably, the decreased complexity and filament length of LHb astrocytes in our *Plch1* deletion model aligns with the hypothesis that astrocytic regulation of neurons is vital for maintaining neural function^61,62^. The reduction in passive astrocytic conductance, potentially mediated by diminished Kir4.1 expression, indicates a disruption in the astrocytes’ ability to buffer extracellular potassium^35^. This disruption may underlie the observed synaptic potentiation and increased neuronal response, probably due to the extracellular potassium accumulation and neuronal depolarization. Such findings underscore a more nuanced role of astrocytes in the tripartite synapse than previously understood, suggesting that not only neuroinflammatory or enhanced alterations in astrocytic morphology and function^63–65^, but also attenuation of astrocytic action could directly influence synaptic dynamics and contribute to the pathophysiology of depression.

An intriguing aspect of our findings is that *Plch1* deletion in astrocytes leads to decreased tonic AMPAR/NMDAR currents and the potentiation of LHb synapses. Conventionally, a reduction of Kir4.1 and its K^+^ buffering action is expected to impair glutamate uptake^66,67^, potentially increasing tonic glutamate levels. However, our observations of decreased tonic currents contradict this expectation. Given that PLCη1 is potentially involved in calcium-signaling pathways, which are critical for gliotransmission, our observation of reduced calcium influx following bradykinin stimulation in our *Plch1* knockdown model may lead to an attenuation of gliotransmitter release and offset the effect of glutamate uptake reduction, resulting in reduced tonic AMPAR/NMDAR currents in LHb neurons. While tonic AMPAR/NMDAR currents are a significant source of extrasynaptic current^55^, our findings suggest that the disruption of tonic currents contributes to the impairment of extrasynaptic LTD previously observed in the LHb^56^ (but see also ref. ^68^, where gliotransmission-derived glutamate is shown to facilitate extrasynaptic LTP). Previous research has linked impaired LTD in the LHb to depressive-like behaviors^56,69^, pinpointing disrupted synaptic homeostasis and skewed neuroplastic balance as a central factor for behavioral changes. Therefore, the potentiation of LHb synapses shown in the astrocytic *Plch1* deletion model may result from a complex interaction where disrupted astrocyte channels in action and gliotransmission-dependent neuroplasticity converge. Further research is required to fully elucidate the intersection of complex mechanisms by which PLCη1 deletion affects synaptic dynamics and neuronal activity in the LHb.

Our findings offer a new perspective on the complex roles of Kir4.1 and GLT-1 in the LHb, particularly in their association with depressive-like behaviors. Previous studies have associated Kir4.1 upregulation with hyperpolarizing LHb neurons, resulting in a shift in neuronal firing mode from tonic to burst, which has been linked to depressive-like states^45^. In our study, we observed a downregulation of Kir4.1 in LHb astrocytes, concurrent with depressive-like behaviors. This seemingly paradoxical finding suggests that the relationship between astrocytic Kir4.1 and depressive-like behaviors is more complex than previously thought. Additionally, our results diverge from the expected agonism of extracellular glutamate associated with decreased LHb GLT-1 expression^58^. We propose that the depressive-like behaviors observed in our PLCη1 cKO model may result from complex interplays between several changes brought by the deletion of PLCη1 in LHb, including morphological alterations in LHb astrocytes, reduced tonic glutamate currents, impaired extrasynaptic LTD and potential disruption of astrocyte calcium signaling. Further studies should aim to elucidate the precise mechanisms through which these complex astrocytic changes influence neuronal function and contribute to the pathophysiology of depression.

Another perspective to consider involves the causative versus compensatory nature of the observed changes in Kir4.1 and GLT-1 expression. These alterations in our specific condition may not be the primary drivers of depressive-like behaviors, but rather secondary responses to altered neuronal dynamics and gliotransmission in the LHb. Elucidating this intricate astrocytic action and its implications would be an integral part of future research to determine the roles and interactions of astrocytic molecules and PLCη1 in LHb and their influence on depressive-like behaviors.

While our investigation has revealed crucial insights regarding the deletion of *Plch1* in astrocytes and its consequential effects on astrocyte morphology, LHb neuronal activity and depressive-like behaviors, certain limitations must be further resolved. Our findings, while indicative, do not fully elucidate the exact molecular pathways through which astrocytic PLCη1 influences neuronal regulation. The observed calcium signaling changes provide clues to the involvement of PLCη1 in neuropeptide signaling pathways. However, a more comprehensive understanding of the role of PLCη1 in the astrocytic regulation of neural activities will require further studies, such as unbiased molecular profiling and functional assays, to dissect the intricate network of interactions in which PLCη1 is involved.

In conclusion, our study emphasizes the importance of PLCη1 in LHb astrocytes, as its deletion led to significant changes in astrocyte morphology, LHb neuronal activity and depressive-like behaviors in mice. Our findings suggest a potential molecular signaling pathway regarding PLCη1, warranting further investigation. Understanding the interactions between PLCη1 and other signaling pathways in LHb astrocytes may contribute to the development of novel treatments for mood disorders.

## Supplementary information

Supplementary information
