# Supplementary material for: Role of phospholipase Cη1 in lateral habenula astrocytes in depressive-like behavior in mice
Source: Exp Mol Med. 2025 Apr 10;57(4):872–87. doi: 10.1038/s12276-025-01432-1 (PMC12046024; doi:10.1038/s12276-025-01432-1)
Supplement: Supplementary file 1 — Supplementary information [file 12276_2025_1432_MOESM1_ESM.docx]

**Role of Phospholipase Cη1 in Lateral Habenula Astrocytes on Depressive-Like Behavior in Mice**

Sukwoon Song^1^†, Miseon Kang^1^†, Jiyoung Lee^1^†, Yong Ryoul Yang^2^, Ho Lee^3^, Jae-Ick Kim^4^, Beomsue Kim^5^, Hoon-seong Choi^6^, Eun-bin Hong^7,8^, Min-ho Nam^7^, Pann-Ghill Suh^9^, Jeongyeon, Kim*^1,10^

**^†^These authors contributed equally to this work.**

**This document includes:**

Supplementary Text

Supplementary Fig. 1 to 7

Supplementary Text

Supplementary methods regarding Supplementary Figure 3

**Primary Astrocyte Culture**

The cerebral cortex C57BL/6J mouse was dissected free of adherent meninges, minced and dissociated into single cell suspension by trituration through a Pasteur pipette. Dissociated cells were plated onto either 12-mm glass coverslips or six-well plates coated with 0.1 mg/mL poly d-lysine (PDL). Cells were grown in Dulbecco’s modified Eagle’s medium (DMEM; Gibco) supplemented with 25 mM glucose, 10% heat-inactivated horse serum, 10% heat-inactivated fetal bovine serum, 2 mM glutamine and 1,000 units/mL penicillin–streptomycin. After 3 days later, cells were vigorously washed with repeated pipetting using medium and the media was replaced to get rid of debris and other floating cell types.

**Calcium imaging**

Astrocytes, transfected with scr-mCherry or shPlch1-mCherry virus for 3–8 h, were incubated with 5 μM Fura-2AM (mixed with 5 μL of 20% Pluronic acid; P3000MP, Invitrogen) for 40 min and washed at room temperature and subsequently transferred to a microscope stage for imagingCultured astrocytes were scanned at 0.5 to 1 frame per second for imaging sessions. A constant flow of fresh buffer perfused the imaging chamber at all times. External solution contained (in mM): 150 NaCl, 10 HEPES, 3 KCl, 2 CaCl2, 2 MgCl2, 5.5 glucose, pH adjusted to pH 7.3. Cells for all the experiments were imaged using a 60X water-immersion objective lens with a numerical aperture of 0.9 and a 488-nm fluorescent imaging filter. Intensity images of 510 nm wavelength were taken at 340 nm and 380 nm excitation wavelengths using iXon EMCCD (DV887 DCS-BV, ANDOR Technology). Two resulting images were used for ratio calculations in Axon Imaging Workbench version 6.2 (Axon Instruments).


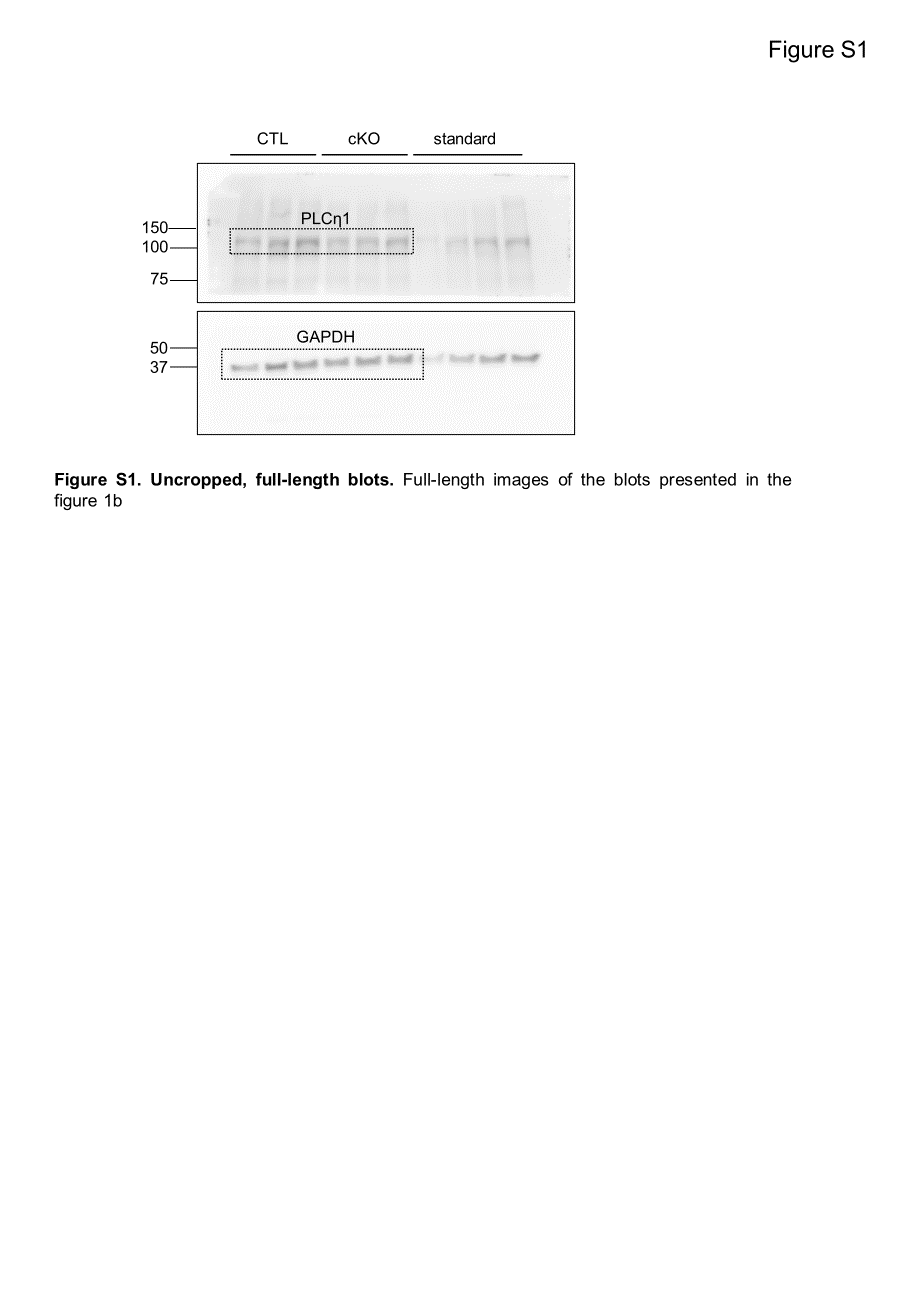


Supplementary Fig. 1. Uncropped, full-length blots. Full-length images of the blots presented in Fig. 1B


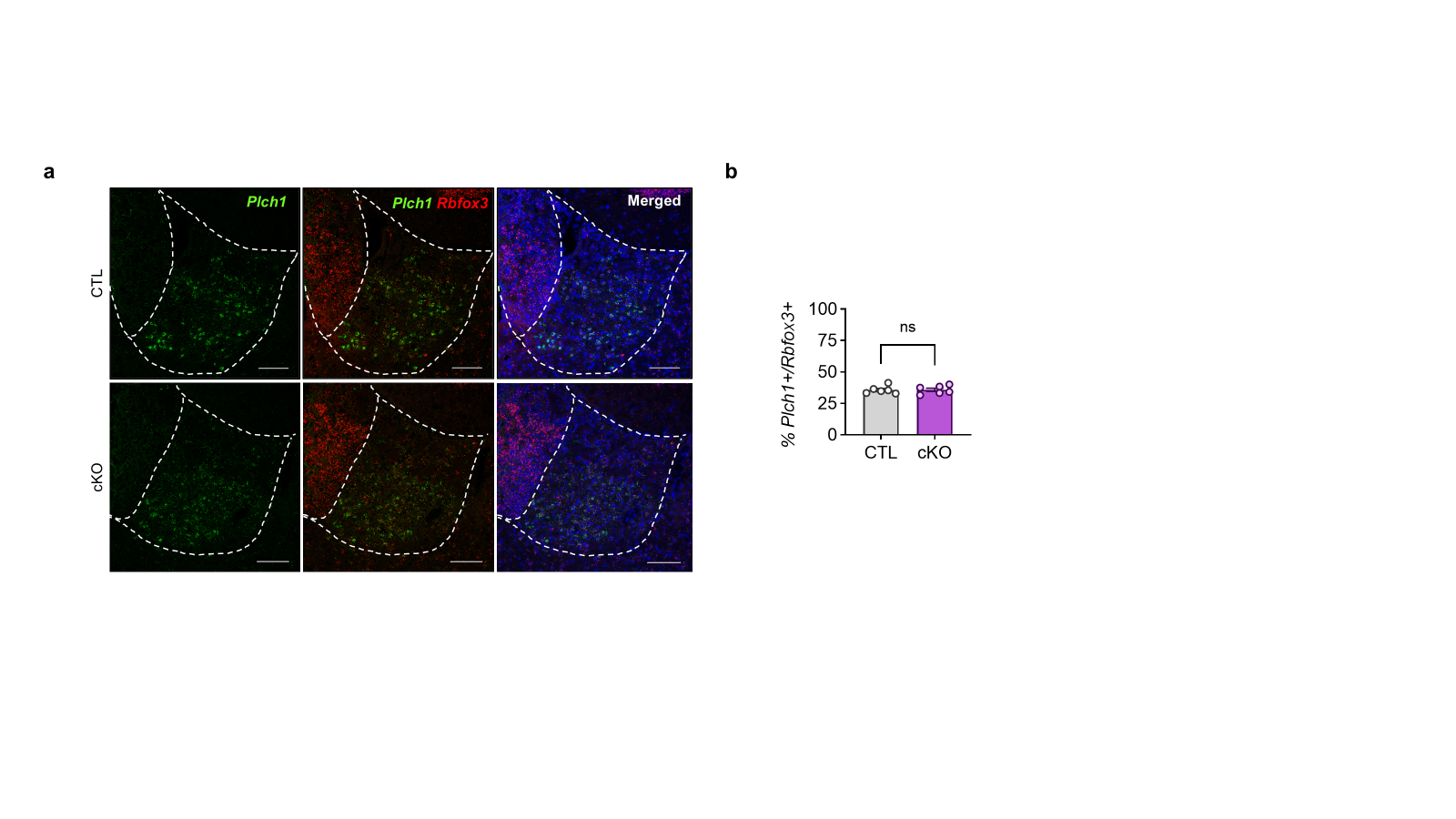


Supplementary Fig. 2. Expression of PLCη1 in Mouse Lateral Habenula Neurons. (a) Representative fluorescent in situ hybridization (FISH) images in the LHb. *Plch1* and the neuronal marker *Rbfox3* (NeuN) were co-labeld. Dashed lines outline the LHb, distinguishing it from the medial habenula (MHb), thalamus, and ventricles. (b) Quantification of neuronal *Plch1* expression in control (CTL) and *Plch1* cKO mice Data are presented as mean ± SEM. The mean values (CTL, 35.76 ± 1.24 %, N = 6; cKO, 35.90 ± 1.35 %, N = 6) show no significant differences between groups (p = 0.94). ‘N’ refers to the total number of animals. Data are presented as mean ± SEM.


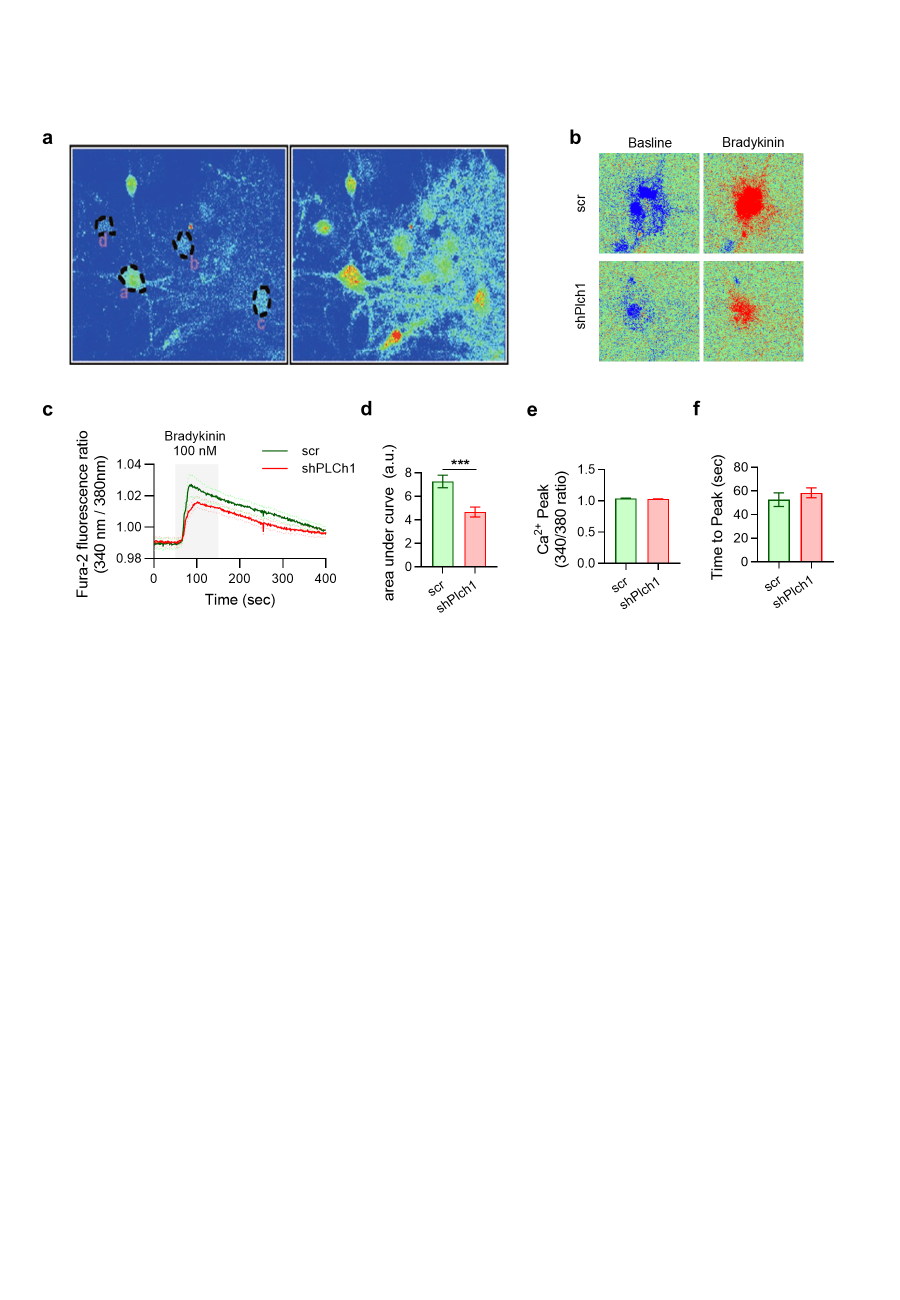


Supplementary Fig. 3. Bradykinin-induced cytosolic calcium response is reduced in shPlch1 transfected cultured astrocyte. Bradykinin-induced cytosolic calcium response is reduced in shPlch1 transfected cultured astrocyte. (a) Representative calcium image of cultured astrocyte. Dashed circle indicates the region of interest. (b) Averaged traces of Bradykinin-induced calcium response of scr and shPlch1 group. (c) The calcium responses were significantly attenuated in the shPlch1 group (n = 56, 103 for scr and shPlch1, Unpaired t-test). (d) There was significant difference between area under curve between scr and shPlch1 injected group but no changes detected in Ca^2+^ peak amplitude (e) and peak dynamics (f). ** indicates p < 0.01.


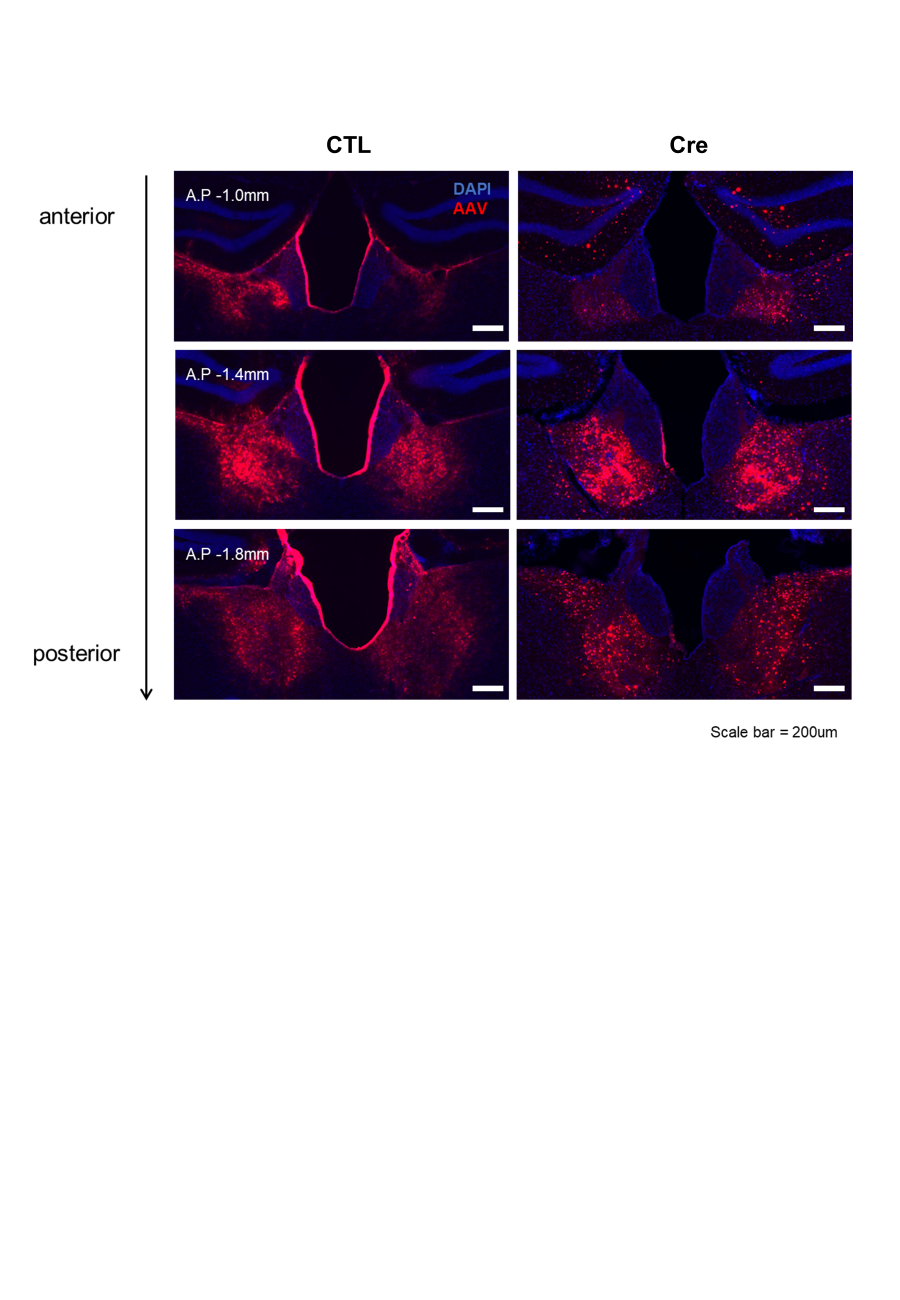


Supplementary Fig. 4. AAV expression in anterior to posterior parts of lateral habenula. The control GFAP-mCherry and GFAP-Cre-mCherry virus vector were injected in the LHb of PLCη1f/f mouse bilaterally. Fluorescent images show that AAV infection anterior to posterior parts of lateral habenula. Blue fluorescents expression shows nuclear and red fluorescents shows AAV. Scale bars = 200um

**
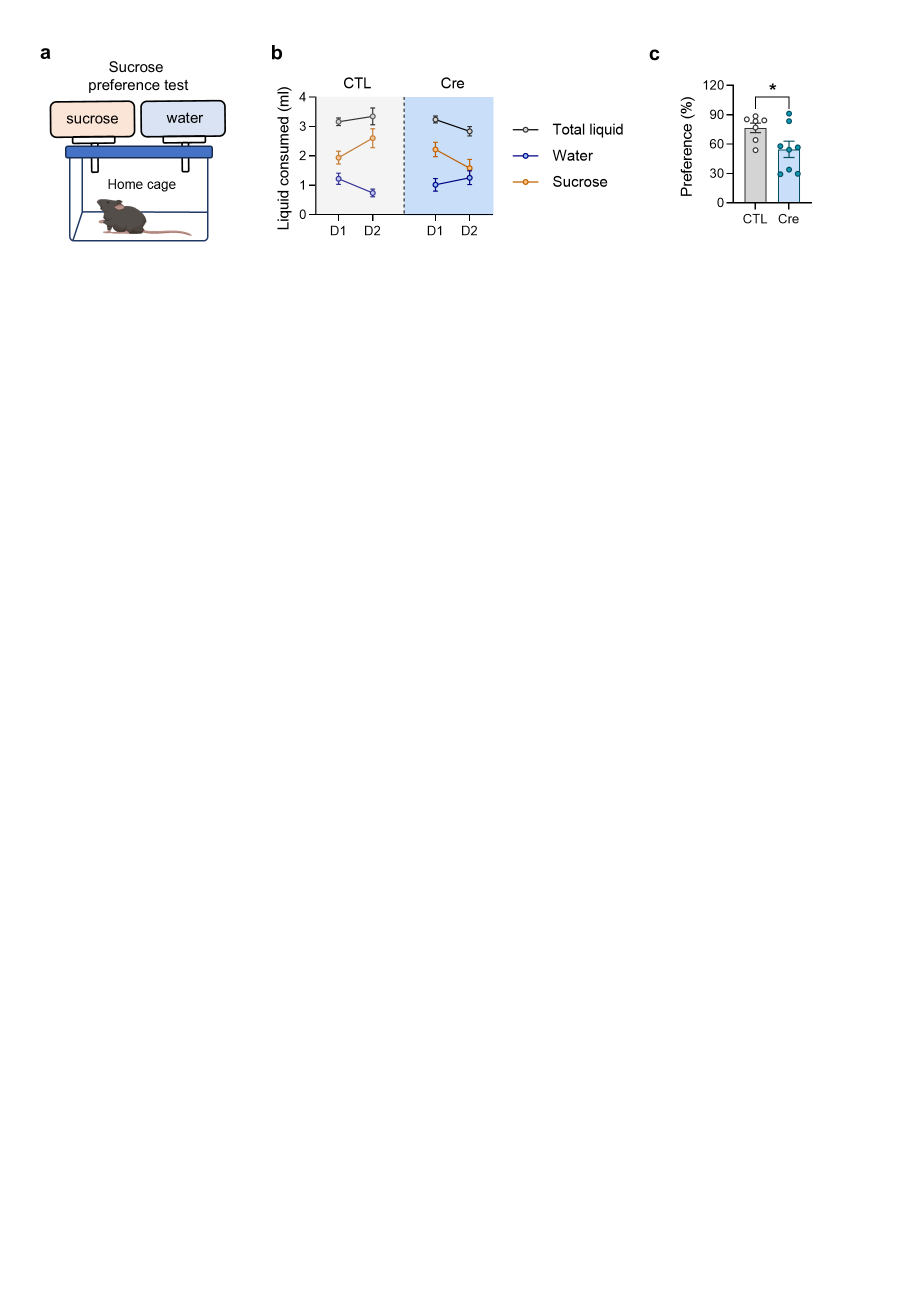
**

Supplementary Fig. 5. Astrocytic PLCη1 deletion in the lateral habenula shows no impact on motor or anxiety-related behaviors. (a) Schematic illustration of the sucrose preference test. (b) Daily consumption tendency of total liquid, 1 % sucrose, water is displayed. CTL shows increased preference on sucrose by D2 compared to D1 while Cre does not. (c) Animals mean daily sucrose consumption per total liquid consumption on D2. CTL, 76.71 ± 4.88 %, *N* = 7; Cre, 54.63 ± 8.32 %, *N* = 8; **p* < 0.05; ‘*N*’ refers total number of animals. Data are presented as mean ± SEM.


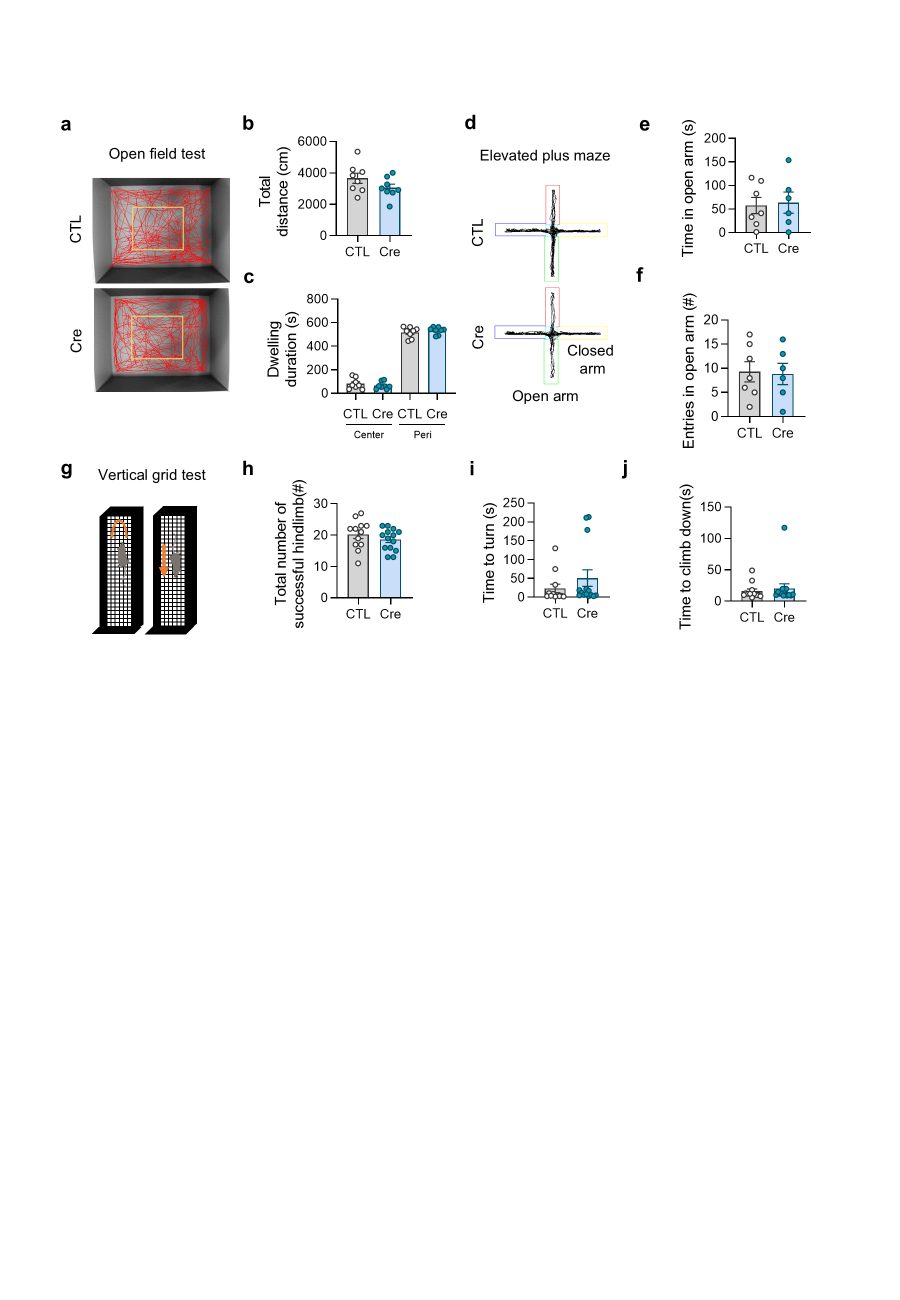


Supplementary Fig. 6. Astrocytic PLCη1 deletion in the lateral habenula shows no impact on motor or anxiety-related behaviors. (a) Representative traces for the open field test (OFT). (b) Bar graph shows that there is no significant differences in total distance moved between groups. (c) Bar graph shows that mice in two groups were not differentiated by the dwelling time either in the center or peripheral region of the arena. (d) Representative trace for the elevated plus maze (EPM) test. (e) The time spent in open arms was not significantly different in two groups. (f) The numbers of open arm entry were not significantly different in two groups. (g) Schematic diagram of the vertical grid test (VGT) setup. (h) Bar graph shows that the total number of successful hindlimb placements during the VGT were not different between groups. (i) Bar graph shows that the latency to turnover during the VGT was not different between groups. (j) Bar graph shows that the total time spent climbing down during the VGT was not different between groups. Data are presented as mean ± SEM.


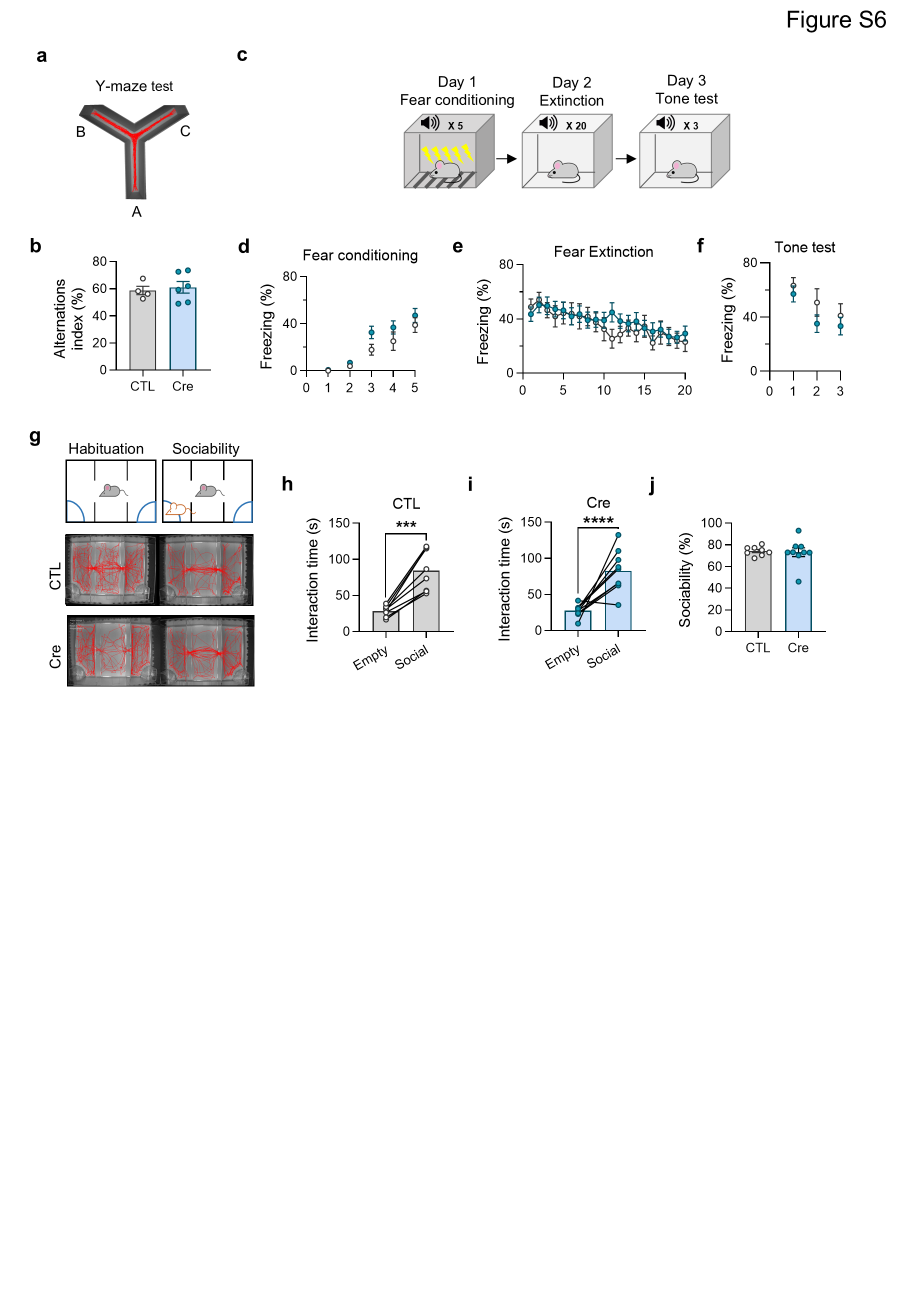


Supplementary Fig. 7. No impairments in motor function, anxiety, or cognitive function. (a) Representative trajectory image of the Y-maze test. (b) Summarized alteration index during Y-maze test displays that there was no significant difference between the control and Cre groups. (c) Schematic diagram of fear memory tests for 3 days. Mice received electrical shocks (0.3 mA, 0.5 sec) paired with pure tone cues (2.8 kHz, 85 dB) for 5 times in day1. the only CS were given on day 2 and the freezing responses to the tone were measured 3 times on day 3 (d-f) Learning curves of conditioned fear during the fear conditioning is not distinguished between CTL and Cre group (g) Schematic illustration of the sociability test (preference on social stimulus over empty cage) and representative mice trajectories during habituation, sociability test. (h, i) Bar graphs of interaction times during sociability test shows that both CTL and Cre groups interact more with the social stimulus than the novel object. (j) Sociability index (interaction time with mouse / (interaction time with object + interaction time with mouse) ✕ 100), displays that the sociability is not significantly different between both groups ***, p< 0.001; ****, p < 0.0001; Data are presented as mean ± SEM.


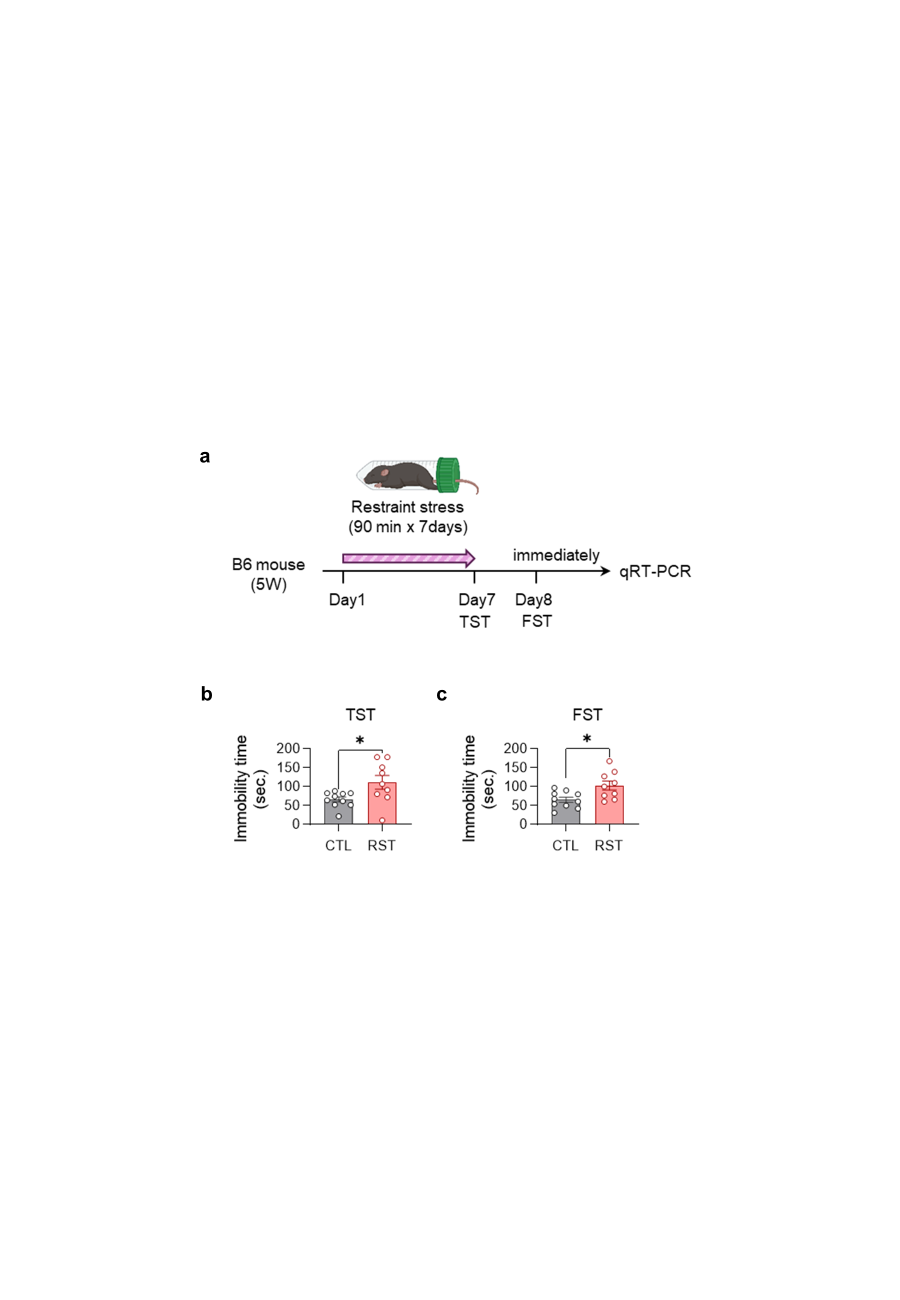


Supplementary Fig. 8. Chronic restraint stress-induced depressive-like behaviors

**(a)** Experimental flow-chart for quantitative Real-Time PCR after chronic stress protocol. **(b, c)** Chronic restraint stress enhanced immobility times between TST (Unpaired t-test, **p* < 0.0255, CTL; 66.16 ± 6.45 sec., *N* = 10, RST; 111.7 ± 18.27 sec., *N* = 9) and FST (Unpaired t-test, *p < 0.0122, CTL; 65.32 ± 6.83 sec., *N* = 10 RST; 103.2 ± 12.05 sec., *N* = 9) tests. Data are presented as mean ± SEM. ‘*N*’ refers to the total number of animals.
